# Genomic Analyses of *Cladophialophora bantiana*, a Major Cause of Cerebral Phaeohyphomycosis Provides Insight into Its Lifestyle, Virulence and Adaption in Host

**DOI:** 10.1371/journal.pone.0161008

**Published:** 2016-08-29

**Authors:** Chee Sian Kuan, Chun Yoong Cham, Gurmit Singh, Su Mei Yew, Yung-Chie Tan, Pei-Sin Chong, Yue Fen Toh, Nadia Atiya, Shiang Ling Na, Kok Wei Lee, Chee-Choong Hoh, Wai-Yan Yee, Kee Peng Ng

**Affiliations:** 1 Department of Medical Microbiology, Faculty of Medicine, University of Malaya, Kuala Lumpur, Malaysia; 2 Department of Neurosurgery, Hospital Pulau Pinang, Jalan Residensi, Georgetown, Pulau Pinang, Malaysia; 3 Department of Surgery, Neurosurgical Division, University of Malaya, Kuala Lumpur, Malaysia; 4 Codon Genomics SB, Selangor Darul Ehsan, Malaysia; University of California Riverside, UNITED STATES

## Abstract

*Cladophialophora bantiana* is a dematiaceous fungus with a predilection for causing central nervous system (CNS) infection manifesting as brain abscess in both immunocompetent and immunocompromised patients. In this paper, we report comprehensive genomic analyses of *C*. *bantiana* isolated from the brain abscess of an immunocompetent man, the first reported case in Malaysia and Southeast Asia. The identity of the fungus was determined using combined morphological analysis and multilocus phylogeny. The draft genome sequence of a neurotrophic fungus, *C*. *bantiana* UM 956 was generated using Illumina sequencing technology to dissect its genetic fundamental and basic biology. The assembled 37.1 Mb genome encodes 12,155 putative coding genes, of which, 1.01% are predicted transposable elements. Its genomic features support its saprophytic lifestyle, renowned for its versatility in decomposing hemicellulose and pectin components. The *C*. *bantiana* UM 956 was also found to carry some important putative genes that engaged in pathogenicity, iron uptake and homeostasis as well as adaptation to various stresses to enable the organism to survive in hostile microenvironment. This wealth of resource will further catalyse more downstream functional studies to provide better understanding on how this fungus can be a successful and persistent pathogen in human.

## Introduction

Phaeohyphomycosis (*photo–Greek for dark*) refers to mycotic infections caused by dematiaceous fungi. These fungi share a common feature of being darkly pigmented due to the presence of dihydroxynaphthalene melanin in their cell walls [1]. Till now, over 150 species and 70 genera of dematiaceous fungi are reported to cause human diseases, ranging from superficial infections to life-threatening infections, such as brain abscess and pulmonary infections [2]. Invasive and systemic phaeohyphomycosis is rare but its causative agents are increasingly recognized as a cause of serious disease such as CNS infections. CNS fungal infections are uncommon as the occurrence is very less and usually associated with devastating consequences in immunocompetent individuals [3]. CNS infections which are caused by fungi can cause one or more the symptoms such as acute or chronic meningitis, encephalitis, abscesses, or myelopathy [4, 5]. *Cryptococcus neoformans* is the predominant causes of cryptococcosis in immunocompetent individuals, while Zygomycetes, *Aspergillus*, and *Candida* species are the most common causes of such infection in immunocompromised patients [6, 7]. Additionally, a few dematiaceous fungi such as *C*. *bantiana*, *Exophiala dermatitidis*, *Ochroconis gallopava* and *Rhinocladiella mackenziei* are recognized as causative agents of primary CNS phaeohyphomycosis [2, 8]. *C*. *bantiana*, *E*. *dermatitidis*, *O*. *gallopava* and *R*. *mackenziei* are known as true neurotropic fungi [9].

Life-threatening CNS fungal infections are commonly associated with an immunocompromised state. Immunocompromised individuals with organ transplantations and acquired immune deficiency syndrome are susceptible to acquire the fungal infection, particularly in brain or meninges. However, primary cerebral phaeohyphomycosis caused by *C*. *bantiana* appears to be an exception to this rule, occurring more commonly in immunocompetent than in immunocompromised patients. Dixon *et al*. [10] used animal experiments to prove that the fungus is carried through bloodstream to the CNS, suggesting that it is disseminated via the haematogenous route to CNS [8, 11, 12]. Amongst the previous cases of cerebral phaeohyphomycosis, *C*. *bantiana* is responsible for causing 48% of the cases and associated with a high mortality rate of up to 70% and there are currently no standardized recommendations for treatment [8]. CNS infections caused by *C*. *bantiana* usually present with brain abscess, either single or multiple lesions [8, 13, 14]. Nonetheless, *C*. *bantiana* rarely causes cutaneous or subcutaneous infections [15].

The advancement and development of sequencing technology and bioinformatics have led to the generation of several neurotropic fungal genomes such as *Cryptococcus gattii* [16] and *C*. *neoformans* [17]. However, at present, the publicly available genome sequence of *C*. *bantiana* remains deficient. Additionally, relatively little is known about molecular mechanisms of pathogenicity and adaptibility of this neurotropic fungus in blood and cerebrospinal fluid (CSF) of the human body. At present, CNS infection caused by *C*. *bantiana* have not been reported in Southeast Asia, although the infections have increasingly been reported abroad [8, 11, 12, 14, 18–21], especially in Europe [12]. In this study, we describe a case of brain abscess caused by *C*. *bantiana* in an immunocompetent man which was successfully treated with surgical excision combined with voriconazole. Additionally, the draft genome of the *C*. *bantiana* was generated by a combined assembly of two different insert-size Illumina sequencing libraries (5-kb insert-size library and 500-bp insert-size library). To our knowledge, this report is the first comprehensive description of the *C*. *bantiana* genome. The thorough analysis of the neurotropic fungal genome will serve as a platform to further understand its basic biology, pathogenicity as well as adaptability in human host.

## Results and Discussion

### Clinical history

A 49-year-old Malay male came with complaints of complex partial seizure, right sided weakness, fever, and headache. A computerized tomography (CT) of the brain showed a rim enhancing lesion over the left parietal region close to the motor strip measuring 4.4 cm (AP) × 2.8 cm (W) × 4.0 cm (H) with mass effect and surrounding edema (S1 Fig). Shortly, he developed slurring of speech, confusion resulting in deterioration of GCS to E1V1M5 (7/15). No history of recent transplantation, trauma and any history suggestive of immunocompromised state was elicited. Inflammatory markers (C-reactive protein and total white cells) were raised. A provisional diagnosis of pyogenic cerebral abscess was made and he underwent an image-guided aspiration of the abscess, in view of its close proximity to the motor strip under cover of intravenous cetriaxone.

A total of 25 mL of viscid, yellow pus was aspirated. Gram and Ziehl-Neelsen smears of the pus did not show any bacteria or acid fast structures. Blood and sputum cultures were sterile. Fungal elements (septate and darkly pigmented fungal hyphae) were observed by a direct microscopic wet mount examination (40% potassium hydroxide). The initial intravenous cetriaxone was changed to empirical intravenous amphotericin B 35 mg OD and oral itraconazole 200 mg BD. The fungal isolate was identified as *C*. *bantiana* based on the typical morphological features and multilocus phylogenetic analysis.

The patient completed a combination of intravenous amphotericin B and oral itraconazole for 45 days after underwent craniotomy and an uneventful excision. He was then discharged home with oral voriconazole for six months. Repeat contrasted CT brain after completion of antifungal treatment showed resolution of the abscess and recovery of motor power of the affected limbs allowing him to perform activities of daily living (power of 4/5).

### Possible transmission route of *C*. *bantiana*

The environmental niche of *C*. *bantiana* is unknown and has seldom been isolated from nature other than nonhuman source. The occupational association of the infection by this neurotropic fungus with farming is suggestive of its origin from this environment as soil fungus [2, 8]. Relatively few reports of its isolation from the environment are on record [22]. A definite relation between an environmental and clinical strain still has to be proven. The patient is a lorry driver, whose payload involves soil, suggesting that soil is the most probable source of his infection, either through a trivial skin wound or inhalation of spores, followed by haematogenous dissemination to the brain. The findings of this case agree with previously reported predisposition of *C*. *bantiana* to people working with soil [8, 23]. Revankar *et al*. (2004) also revealed many cases of immunocompetent patients occur in rural settings, possibly due to more prevalent soil exposure [8].

### Morphological characterization of *C*. *bantiana* UM 956

**Fig 1:**
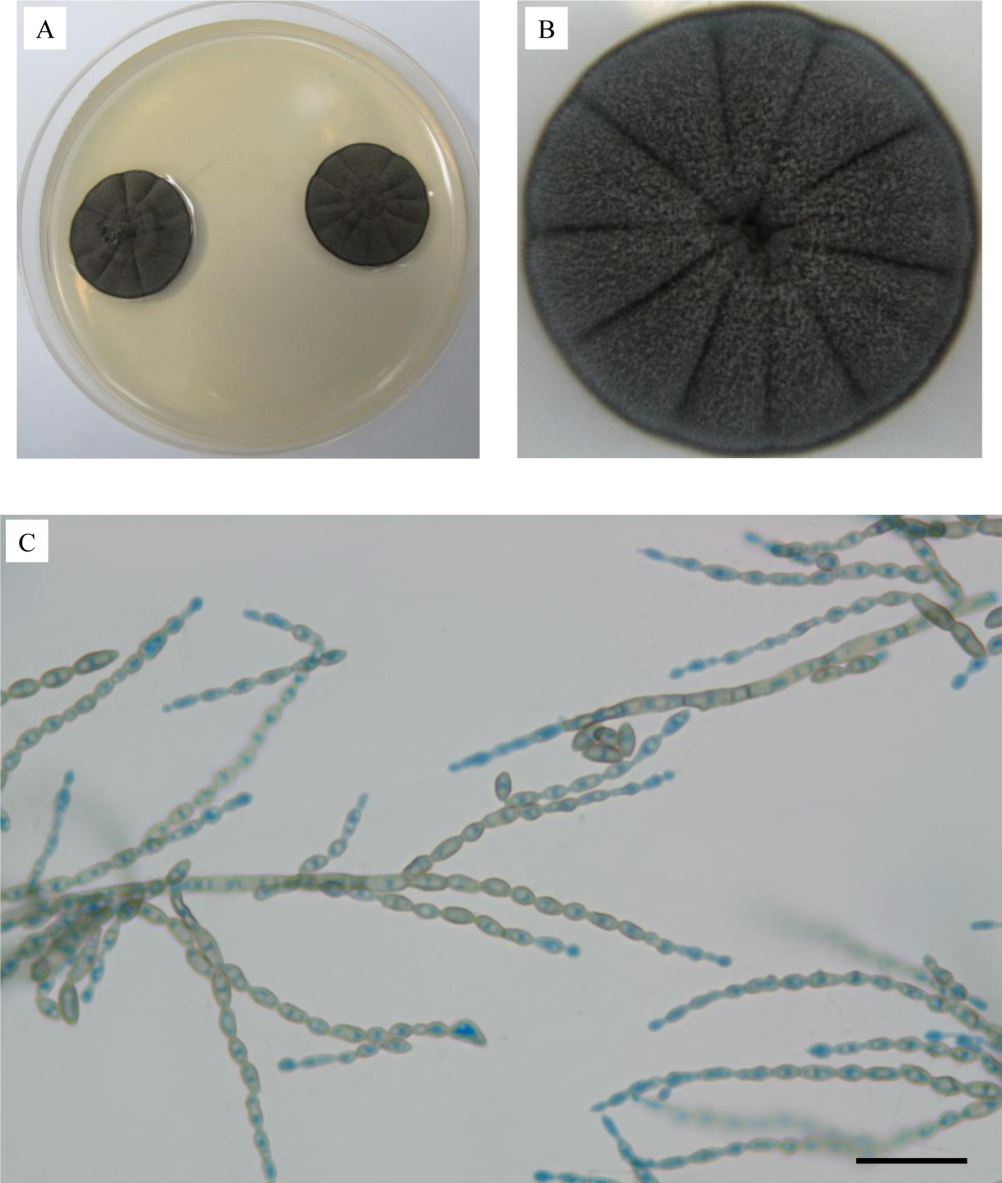
Colonial characteristic and microscopic morphology of *C*. *bantiana*. The (A) surface and (B) close-up view of the colonial morphology of *C*. *bantiana* after being cultured for seven days. Light micrograph showing (C) smooth walled, pale olivaceous, ellipsoidal to spindle-shaped conidia arranged in long, strongly coherent chains (400× magnification, bars 20 μm).

The growth rate of *C*. *bantiana* UM 956 on SDA was moderate and it takes around 2–3 days of incubation at 30°C to observe visible growth. The colonies mature and reached diameters of 30 mm after seven days of incubation at 30°C. The *C*. *bantiana* UM 956 colonies were compact, velvety textured and olivaceous to brown discolouration with a black undersurface (Fig 1A and 1B). The fungus did not produce any diffusible pigment. Microscopic examination with lactophenol cotton blue staining showed dark septate hyphae with sparsely branched conidiophores producing long, strongly coherent and wavy chains of conidia (Fig 1C). The conidia (1–5 × 1–2 μm) were pale olivaceous, ellipsoidal to spindle-shaped (Fig 1C). No chlamydospores were observed. On the basis of these characteristics, the isolate was found to be morphologically related to *C*. *bantiana* [24].

### Sequence-based identification and multilocus phylogenetic analysis

The preliminary morphological identification of the UM 956 isolate was confirmed by PCR amplification of the ITS, SSU and LSU gene regions, followed by BLASTn search against those (ITS, SSU, and LSU nucleotide sequences) deposited in the NCBI-nucleotide database. The ITS sequence of UM 956 showed 100% (558/558) identical to the *C*. *bantiana* strain PWQ2235 isolate ISHAM-ITS_ID MITS1144. However, the SSU and LSU were 92% (925/1005) and 93% (1367/1470 bp) identical to the *Chaetothyriales* sp. TRN486 and the *Cladophialophora carrionii* isolate CBS 160.54, respectively.

**Fig 2:**
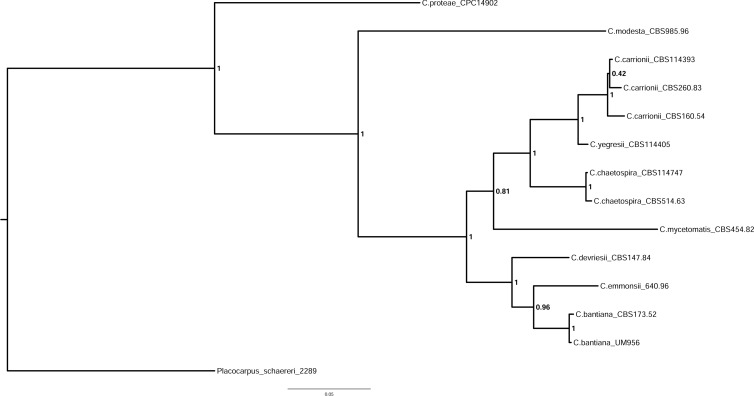
Bayesian phylogram generated using the combined gene sequences of ITS and LSU. The tree was rooted with *Plococarpus schaereri* AFTOL-ID 2289 as outgroup. The numbers on the nodes indicate Bayesian posterior probability based on 100 sampling frequencies for a total of 100,000 generations.

Multilocus phylogenetic analysis was subsequently used to identify UM 956 to the species-level. The sequenced ITS and LSU gene regions were used to construct a phylogram using combined gene analysis with an additional 12 ex-type strains of the *Cladophialophora* species (Table 1). The SSU gene region was excluded in the multilocus phylogenetic analysis due to the poor alignment between UM 956 and other *Cladophialophora* species (including other strains of *C*. *bantiana*). The multilocus phylogenetic tree consisted of members from the genus *Cladophialophora* and the *Cladophialophora* species are well separated (Fig 2). The analysis revealed that UM 956 was tightly clustered together with *C*. *bantiana* type strain CBS 173.52 (Fig 2). The high statistical support (1.0 Bayesian posterior probability) for the placement of UM 956 with *C*. *bantiana* type strain CBS 173.52 confirms its identity as *C*. *bantiana*.

**Table 1:** Details of Isolates Subjected to Multilocus Phylogenetic Analysis.

ITS	LSU	Organism name	Strain	Origin	Country
KU928131	KU928133	*Cladophialophora bantiana*	UM 956	Brain abscess	Malaysia
EU103989	KF155189	*Cladophialophora bantiana*	CBS 173.52	Brain abscess	USA
EU103995	KC809995	*Cladophialophora emmonsii*	CBS 640.96	Sub-cutaneous lesion, cat	Netherlands
EU103985	KC809989	*Cladophialophora devriesii*	CBS 147.84	Disseminated infection	USA
EU137293	KC809991	*Cladophialophora mycetomatis*	CBS 454.82	Culture contaminant	Netherlands
EU035406	KF928513	*Cladophialophora chaetospira*	CBS 514.6	Wheat field soil	Germany
EU035403	KF928514	*Cladophialophora chaetospira*	CBS 114747	*Phyllostachys bambusoides*	China
EU137322	KC809994	*Cladophialophora yegresii*	CBS 114405	*Stenocereus griseus*	Venezuela
GU225939	KF928485	*Cladophialophora modesta*	CBS 985.96	Brain	USA
FJ372388	FJ372405	*Cladophialophora proteae*	CPC 14902	*Encephalartos altensteinii*	South Africa
EU137292	KF928518	*Cladophialophora carrioni*	CBS 260.83	Skin lesion	USA
EU137266	KF928517	*Cladophialophora carrioni*	CBS 160.54	Chromoblastomycosis	Australia
EU137268	KF928516	*Cladophialophora carrioni*	CBS 114393	Chromoblastomycosis hand lesion	Venezuela
-	EF643766	*Placocarpus schaereri*	AFTOL-ID 2289	-	USA

### Acquisition of the *C*. *bantiana* UM 956 genome sequence

The genome of *C*. *bantiana* UM 956, was sequenced using Illumina HiSeq 2000 system. A total of 28,594,774 paired reads (2.57 Gb) of a 500-bp insert-size library and 12,972,712 paired reads (1.17 Gb) of a 5-kb insert-size library were generated by Illumina HiSeq 2000 Sequencing system. The sequencing coverage for the combined sequenced reads is 90-fold. The total assembly size of the genome is 37.1 Mb. The draft genome sequence consists of 439 contigs (≥ 200 bp) and orientated within 84 scaffolds (≥1,000 bp) (Table 2). The genome had an average GC content of 50.65%. A summary of the principal genome sequence data is provided in Table 2.

**Table 2:** *C*. *bantiana* UM 956 genomic and assembly features.

Details	Paired-end and mate pair combined (500-bp and 5-kb)
Sequencing depth	~90×
Total length of sequences (bp)	37,087,895
Total number of contigs (≥200 bp)	439
Contigs N50 (bp)	172,990
Contigs GC content (%)	51.27
Total number of scaffolds (≥1,000 bp)	84
Scaffolds N50 (bp)	3,618,913
Scaffolds GC content (%)	51.19
tRNAs	58
8s rRNA	17
16s rRNA	1
28s rRNA	1
Number of predicted genes (≥ 99bp)	12,155
Annotated protein coding regions (nr)	10,543
Annotated protein coding regions (SwissProt)	1,464
Annotated protein coding regions (Interpro)	9,324
Hypothetical proteins	8,444

### Gene content

A total of 58 tRNAs and 19 rRNAs were identified in the UM 956 genome. Within the genome, a total of 12,155 putative genes (≥ 33 amino acids) were predicted. These putative genes consist of 48.5% of the assembly (1 gene per 3.1 kb). On average, there are 2.25 exons per gene and average size of protein coding genes is 1,481 bp in the genome. The basic genomic statistics, assembly size and the total number of predicted genes in the *C*. *bantiana* UM 956 genome were compared to other pathogenic dematiaceous fungi (Table 3, S1 Table). The genome size of *C*. *bantiana* UM 956, number of putative genes and gene density are comparable to the recently sequenced dematiaceous fungi (Table 3).

**Table 3:** Genome content of *C*. *bantiana* UM 956 and other previously sequenced fungal genomes.

Species	Assembly size (Mb)	Predicted genes	Gene density (gene/10 kb)	GC content (%)	Isolation site	Reference
*C*. *bantiana* UM 956	37.1	12,155	3.3	51.27	Brain abscess	In this study
*Bipolaris papendorfii* UM 226	33.4	11,015	3.29	50.65	Skin scraping	[26]
*Daldinia eschscholtzii* UM 1020	35.5	11,120	3.1	46.81	Blood	[27]
*Daldinia eschscholtzii* UM 1400	35.8	10,822	3.0	46.8	Skin scraping	[27]
*Ochroconis mirabilis* UM 578	34.6	13,435	3.88	52.1	Skin scraping	[28]
*Pyrenochaeta* sp. UM 256	35.5	12,545	3.53	50.4	Skin scraping	[29]
*Sporothrix schenckii* strain 1099–18	32.4	10,293	3.17	54.96	Subcutaneous tissue	[30]
*Exophiala dermatitidis* NIH/UT8656	26.4	9269	3.51	51.51	Unknown	[31]

A total of 10,543, 1,464, and 9,324 gene-coding sequences are homologous to known proteins in the NCBI nr, SwissProt, and InterPro databases, respectively (S2 Table). A total of 8,444 hypothetical proteins (50% identity and 70% coverage cut-off) were identified based on the top hit of the BLAST result against NCBI nr database. We found that more than 95% (8085 sequences) of these hypothetical proteins hits to proteins sequences from *C*. *psammophila* CBS110553. InterPro protein sequence analysis revealed at least one domain was predicted in 6,527 hypothetical proteins in *C*. *bantiana* UM 956 (S3 Table). It is noted that *C*. *bantiana* UM 956 has 150 hypothetical proteins engaged in cytochrome P450 (CYP) superfamily. These enzymes perform various reactions in a wide variety of physiological processes, such as biosynthesis of secondary metabolites, detoxification, and degradation of xenobiotics [25].

**Fig 3:**
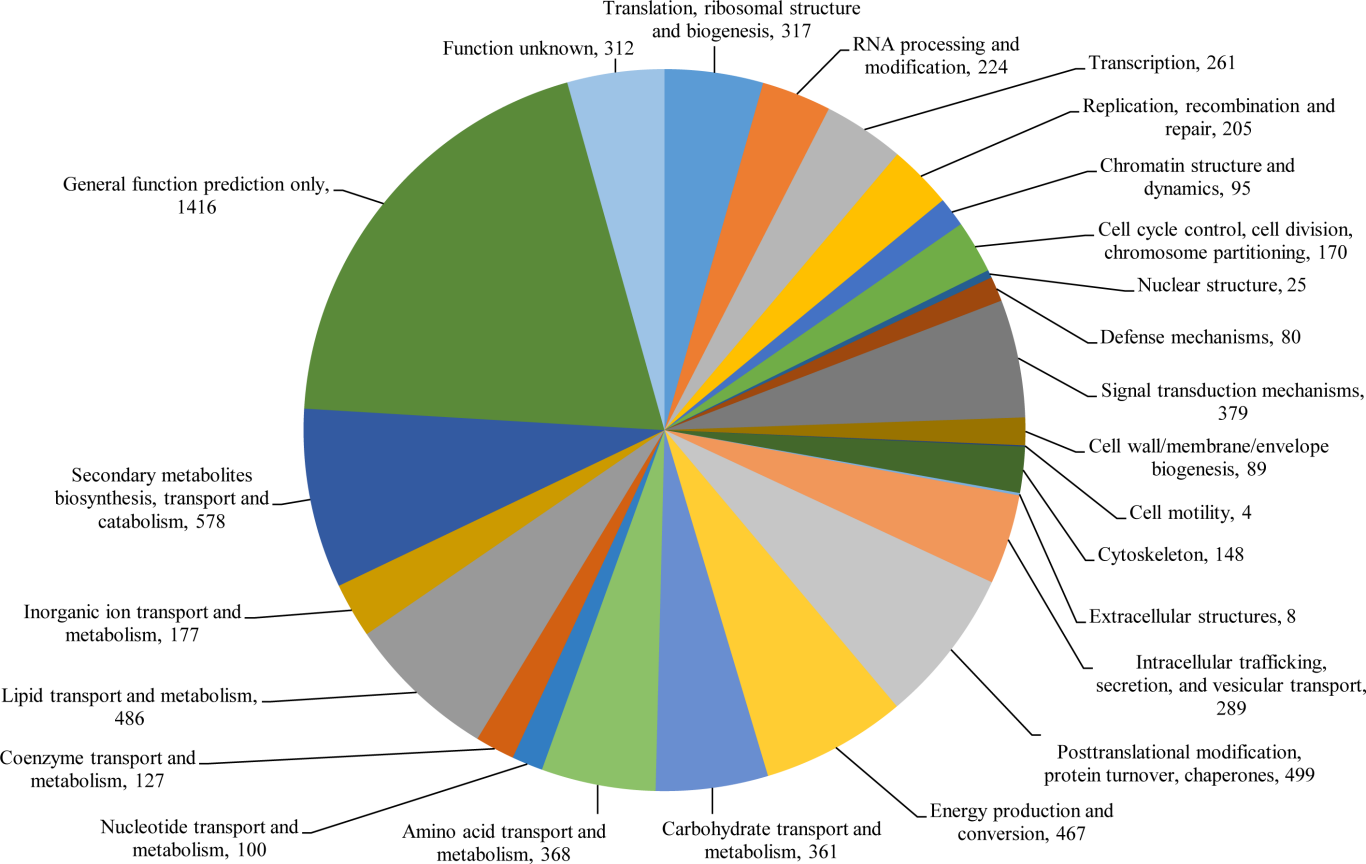
KOG class annotation distribution of *C*. *bantiana* UM 956 genome. A total of 7,185 of protein-coding genes were annotated by KOG. The proteins were assigned into different KOG functionary categories as shown in the pie chart.

The genome was further mapped to the Eukaryotic Clusters of Orthologs (KOG) database to further characterize the putative proteins. A total of 7,185 (59.1% of total predicted genes) of protein-coding genes were mapped in the KOG database and were classified to 26 different functional groups (Fig 3 and S4 Table). The UM 956 genome contains large amount of putative genes (1,728 genes) that were not categorized to a distinct group (categories “General functions prediction only” and “Function unknown”). Apart from the poorly characterized categories: categories “General functions prediction only” and “Function unknown”, the top five most abundant KOG groups were “Secondary metabolites biosynthesis, transport and catabolism” (578 genes), “Post-translational modification, protein turnover, chaperones” (499 genes), “Lipid transport and metabolism” (486 genes), “Energy production and conversion” (467 genes), and “Amino acid transport and metabolism” (368 genes). In contrast to other dematiaceous fungi, such as *B*. *papendorfii* UM 226 (334 genes), *Pyrenochaeta* sp. UM 256 (395 genes), *Ochroconis mirabilis* UM 578 (431 genes), *D*. *eschscholtzii* UM 1020 (358 genes), and *D*. *eschscholtzii* UM 1400 (356 genes), the predominant of the functionally annotated genes in UM 956 genome was engaged in biosynthesis and catabolism of secondary metabolites. The metabolism and biosynthesis of secondary metabolites in *C*. *bantiana* UM 956 are further discussed in the subsection of “Secondary metabolism”.

### Transposable elements

Genomic plasticity allows organisms to survive and adapt to diverse environments, which is particularly related to pathogens to engage in novel niche. Transposable elements (TEs) have been proposed to actively promote genomic rearrangements [32], although their exact role in the evolution genomic DNA remain unknown. Dematiaceous fungal genomes contain greatly different amounts of TEs. *B*. *papendorfii* UM 226, *Cochliobolus heterostrophus* race O genome, *Sporothrix schenckii*, *Sporothrix brasiliensis*, *O*. *mirabilis* UM 578, *D*. *eschscholtzii* strains UM 1400 and UM 1020 contain 2.49%, 5.9%, 0.34%, 0.62%, 0.01%, 1.02%, and 1.42%, respectively [26, 27, 30, 33, 34]. In this study, the TEs occupy 1.01% (374,655 bp) of the *C*. *bantiana* UM 956 genome (Table 4). The class I, retrotransposons comprised 0.94% of the genome, whereas class II transposons, DNA transposons comprised only 0.07% of the genome. As previously noted in dematiaceous fungal pathogens [26, 30, 34], LTR (Long Terminal Repeat) is the major type of retrotransposons, in which the Gypsy-like element is more than Copia-like element. Our bioinformatics prediction revealed that 0.86% and 0.0058% of the sequenced UM 956 genome consists of gypsy and TY1_Copia, respectively. Furthermore, one copy of piggyBac transposable element was identified in UM 956. This transposable element was isolated from the cabbage looper moth *Trichoplusia ni*. PiggyBac transposon has been used as transposon-based mutagenesis tool for fission yeast [35] as well as for the genetic characterization of mammalian genomes [36–38].

**Table 4:** Putative transposable elements in the genome sequence of *C*. *bantiana* UM 956.

Class	Family name	Total number	Total bases	Percentage of assembled genome
I	DDE_1	24	19,761	0.0533%
	gypsy	173	319,870	0.8625%
	LINE	8	5596	0.0151%
	TY1_Copia	5	2164	0.0058%
II	helitronORF	2	1363	0.0037%
	hAT	8	10657	0.0287%
	mariner	12	10680	0.0288%
	mariner_ant1	3	1587	0.0043%
	MuDR_A_B	3	1599	0.0043%
	cacta	1	89	0.0002%
	piggybac	1	1289	0.0035%
	Total	240	374,655	1.01%

### KEGG pathway analysis of *C*. *bantiana* UM 956

**Fig 4:**
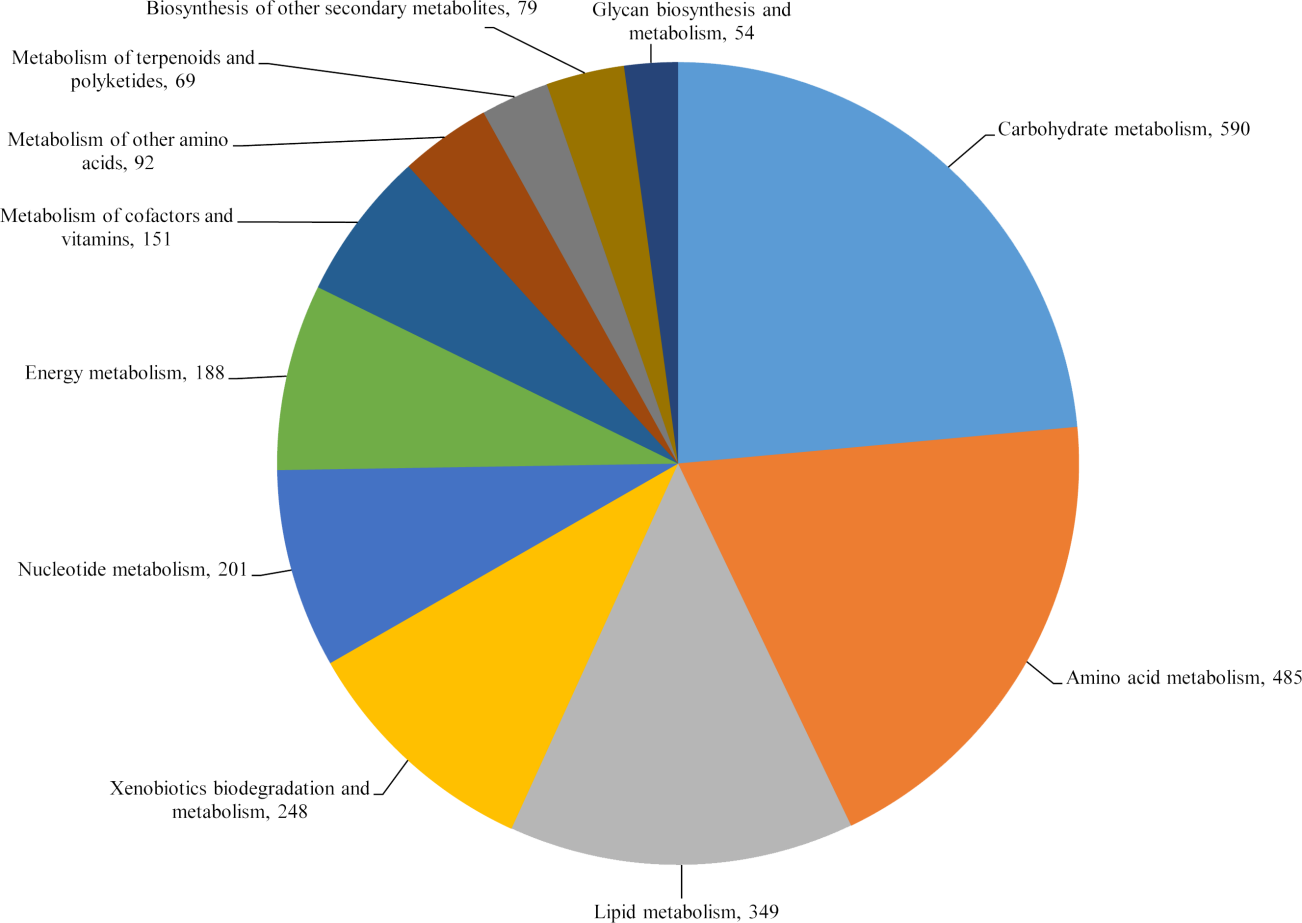
KEGG classifications of proteins in *C*. *bantiana* UM 956 genome. The proteins were assigned into different KEGG metabolic pathway categories as shown in the pie chart. A total of 2,506 protein-coding genes were involved in metabolic pathway based on the KEGG database.

KEGG pathway analysis was carried out to further gain insight into the gene functions in *C*. *bantiana* UM 956. A total of 2,506 predicted proteins was assigned to their orthologous genes in metabolic pathways in the KEGG database. The full list of KEGG pathway annotation was listed in S5 Table. The top five categories in KEGG metabolic pathway are carbohydrate metabolism, amino acid metabolism, lipid metabolism, xenobiotics biodegradation and metabolism, and nucleotide metabolism (Fig 4). In particular, there are 590 unique reactions corresponding to carbohydrate metabolism. *C*. *bantiana* UM 956 contains many enzymes involved in glycolysis, gluconeogenesis, citrate cycle (TCA cycle), and other essential carbohydrate metabolism pathways, which suggest this fungus is capable in metabolize glucose, sucrose, galactose, fructose, mannose pyruvate, and starch (S5 Table). The potential of using sugars other than glucose as well as sulfur, nitrogen, and peptide nitrogen sources may partly explain the ubiquitous nature of *C*. *bantiana* in the environment.

### Carbohydrate-active enzymes

The plant cell wall is composed of polysaccharides cellulose, hemicellulose, and pectin, which is important as a nutrient source for plant pathogens and saprophytes and act as physical barrier to plant pathogens. Fungi can produce diverse carbohydrate-active enzymes (CAZymes) to degrade enormous functional and structural diversity of complex plant polysaccharide materials for carbon sources [39]. Thus, CAZymes can be powerful reporters of the fungal lifestyle. *C*. *bantiana* has been reported widely distributed in soil and woody plant materials and readily as saprophyte, although the precise niche of the fungus remains unidentified [8, 22, 40]. As a matter of fact, saprophytes degrade plant cell wall materials to obtain nutrients for growth. In this work, we have probed the CAZyme repertoires of the *C*. *bantiana* UM 956 and compared with other 12 fungi of different lifestyles. Differences in the number and distribution of CAZymes among *C*. *bantiana* UM 956, non-plant pathogens (biotrophic, saprophytic, symbiotic fungi), and plant pathogens (necrotrophic, hemi-biotrophic, and facultative parasitic fungi) were analyzed.

**Fig 5:**
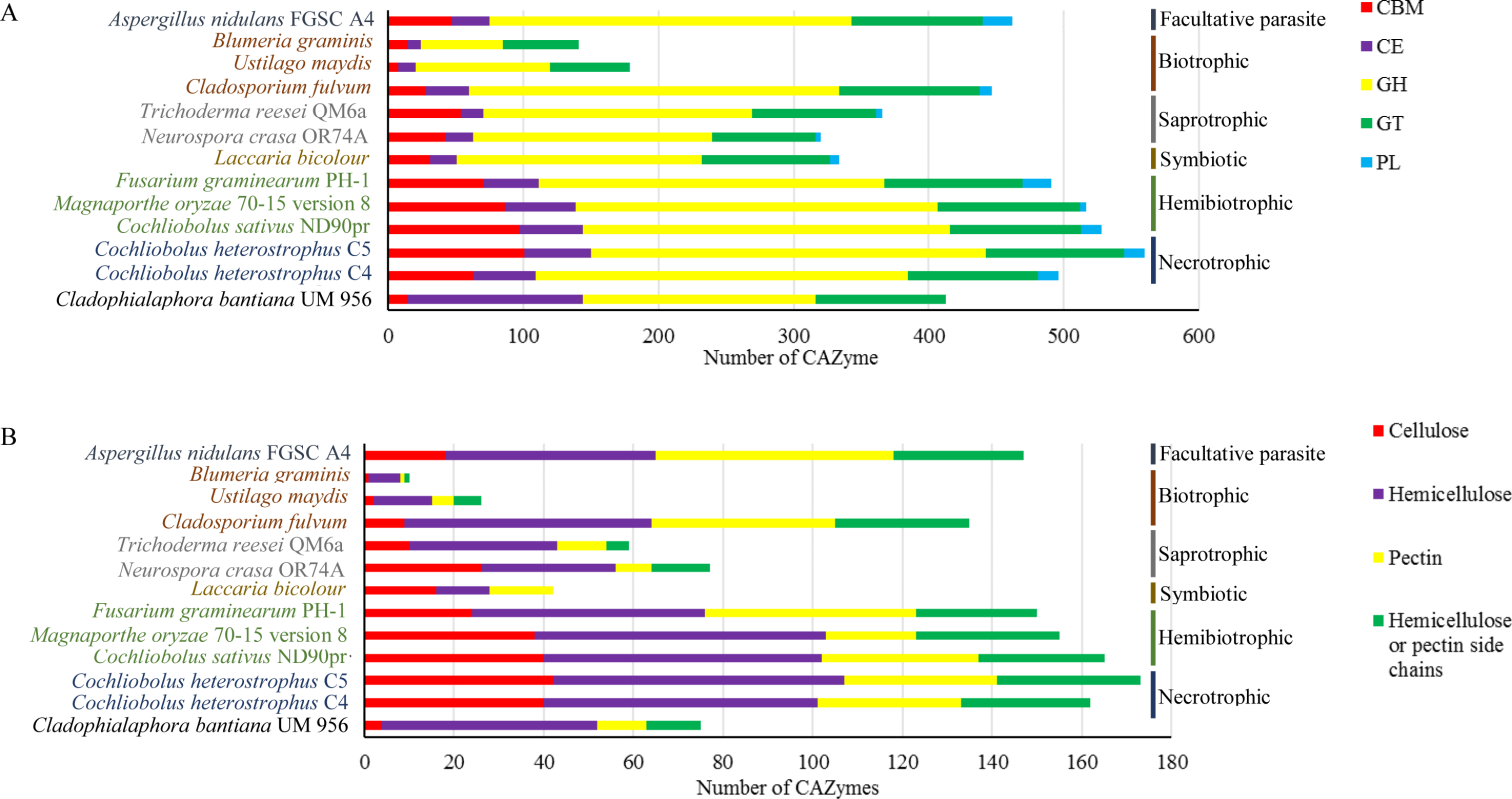
CAZyme class annotation distribution of *C*. *bantiana* UM 956 genome. (A) Comparison of the distribution of CAZyme catalytic domains between *C*. *bantiana* and fungi from various lifestyles. (B) Comparison of the plant cell wall degrading potential from CAZyme analysis between *C*. *bantiana* and fungi from various lifestyles. AA: auxiliary activities; CBM: carbohydrate binding module; CE: carbohydrate esterase; GH: glycoside hydrolase; GT: glycosyltransferase and PL, polysaccharide lyase.

A total of 484 genes encoding putative CAZymes, comprising 14 carbohydrate binding module (CBM), 130 carbohydrate esterases (CE), 172 glycoside hydrolases (GH), and 97 glycosyl transferases (GT) (S6 Table). In general, *C*. *bantiana* UM 956 has fewer CAZymes than plant pathogenic fungi but comparable to saprotroph and symbiotic fungi (Fig 5A). The CAZyme content in UM 956 is larger than in biotrophic fungi, except for *Cladosporium fulvum*, renowned for its capability in degrading plant cell wall materials [39]. No polysaccharide lyase (PL) was identified in the *C*. *bantiana* genome. Zhao *et al*. [39] revealed that most of the fungi that lack of PL are saprophytic fungi. Moreover, saprophytic fungi tends to loss CE7, CE8, CE11, GH6, GH11, GH73, GH80 and GH82 families [39], which is also observed in *C*. *bantiana* genome. Although experimental supports were deficient, the absence of these CAZyme families may be correlated to the saprotrophic lifestyle of *C*. *bantiana*.

The *C*. *bantiana* UM 956 genome contains 75 CAZymes unambiguously related to the breakdown of plant cell wall polysaccharides, such as cellulose, hemicellulose, pectin, and hemicellulose or pectin side chains (S7 Table). As shown in Fig 5B, the number of plant cell wall degrading enzymes in UM 956 was noticeably lower than necrotrophic and hemi-biotrophic fungi but equivalent in size to the saprotrophic fungi. Even if UM 956 displays the quantitative similarity to the saprotrophic fungi, but this neurotropic fungus shows qualitative differences to those of saprobes (Fig 5B and S7 Table). It is conspicuous that the reduction in genes encoding cellulose degrading CAZyme in *C*. *bantiana* is extreme (Fig 5B and S7 Table). The cellulose degrading capacity for *C*. *bantiana* is significantly smaller than necrotrophic, hemi-biotrophic, facultative parasitic, saprotrophic, and symbiotic fungi, but, not far-off to biotrophic fungi, *Ustilago maydis* and *Blumeria graminis*. In contrast, its potential hemicellulose and pectin degrading capacity are strikingly similar to that of *Neurospora crassa* and *Trichoderma reesei*. The CAZyme content in the *C*. *bantiana* genome suggests a preference of this fungus for hemicellulose and pectin rather than cellulose.

Among the unique characteristics of the *C*. *bantiana* genome is the presence of an abundant CE1 proteins (31 CE1 proteins), whereas saprotrophic fungi have an average of four genes (minimum 2, maximum 6) (S7 Table). The CE1 family includes feruloyl esterase, acetyl xylan esterase, cinnamoyl esterase, carboxylesterase, S-formylglutathione hydrolase, diacylglycerol O-acyltransferase, and trehalose 6-O-mycolyltransferase. The members of GH61 family are copper-dependent lytic polysaccharide monooxygenases that degrade lignocellulose by catalysing the oxidative cleavage of cellulose [41]. Nonetheless, the GH61 was not determined in *C*. *bantiana*, whereas *N*. *crassa* and *T*. *reesei* have respective three and two genes. In particular, *C*. *bantiana* lacks most of the CAZyme families correlated with the plant biomass degradation, including CE5, GH6, GH7, GH36, GH53, GH54, GH62, PL1, and PL3 (S7 Table). Our data showed that UM 956 decompose plant cell wall polysaccharides in a manner strikingly different from the saprotrophic fungi. The number of cellulose degrading enzymes encoded in *C*. *bantiana* is smaller than *N*. *crassa* and *T*. *reesei*, indicative of a lower preference to cope with plant cellulose tissue. All these characteristics relate well to a saprotrophic lifestyle that utilizes plant biomass that has been pre-digested by previous colonizers or employs different approaches to degrade plant polysaccharides.

### Secreted peptidases

One of the features of saprotrophic lifestyle is the lack of predominance of a specific protease family. In fact, they use a wide variety of extracellular proteases to degrade different types of substrate complexes in their environmental niches, indicative their less specialized nutritional status [42–44]. This observation is in agreement with our data that *C*. *bantiana* produces broad spectrum of protein degrading enzymes. Peptidase-encoding genes were categorized in the predicted proteomes of the *C*. *bantiana* UM 956 genome using the MEROPS database, with emphasis on the extracellular peptidases. We identified 136 peptidases in *C*. *bantiana* UM 956, of which, 19 were extracellular peptidases (nine serine peptidases, six aspartic peptidases, two metallopeptidases, one cysteine peptidase, and one threonine peptidase) (S8 Table).

Cellular invasive processes involve the degradation of extracellular matrix and basement membranes by different extracellular proteases to enable microbial cells to spread through anatomic barriers to migrate into circulation [45]. Several studies indicated that metalloproteases belong to the M35 and M36 families are expanded in fungal pathogens as adaptation to human or animal hosts [46, 47]. Vu *et al*. [48] reveled that a secreted fungalysins (M36), known as Mpr1, play a vital role for *C*. *neoformans* to breach the endothelium of human blood-brain barrier (BBB) to establish fungal CNS disease [48]. However, we have not observed any deuterolysins (M35) and fungalysins (M36) in the neurotropic *C*. *bantiana* UM 956, suggesting it employs other approaches to cause fungal CNS infection. *C*. *bantiana* UM 956 was predicted to secrete a repertoire of different endo- and exopeptidases, including the peptidases of MEROPS subfamilies A01, S08, S09, S10, and M28. These extracellular peptidases were secreted by pathogenic fungi to digest proteins in hostile environments of the extracellular matrix with optimal activity at low pH [49]. Pathogenic *Candida* species comprise a gene family coding for candidapepsin (SAPs) which are virulence factors for localized and disseminated candida infections [50, 51]. The expression of SAPs are associated with hyphal formation, overcome the host immune system and adherence to numerous host tissues and cell types by digestion of host surface proteins [52]. In this study, we identified a putative secreted candidapepsin, SAP3 (UM956_10742) in *C*. *bantiana* UM 956 genome. The expression of SAP3 is associated with oral disease and vaginal infection [53]. Exhaustive experiments have been performed to reveal that the SAP3 is one of the principal *C*. *albicans* aspartyl proteinases that is involved in mucosal adherence for establishing mucocutaneous infections [54]. Sap3 is expressed during early stages of epithelial colonization and subsequently during mucosal tissue damage [52, 54–56], suggesting its adherence and invasive properties in fungal pathogenicity.

**Fig 6:**
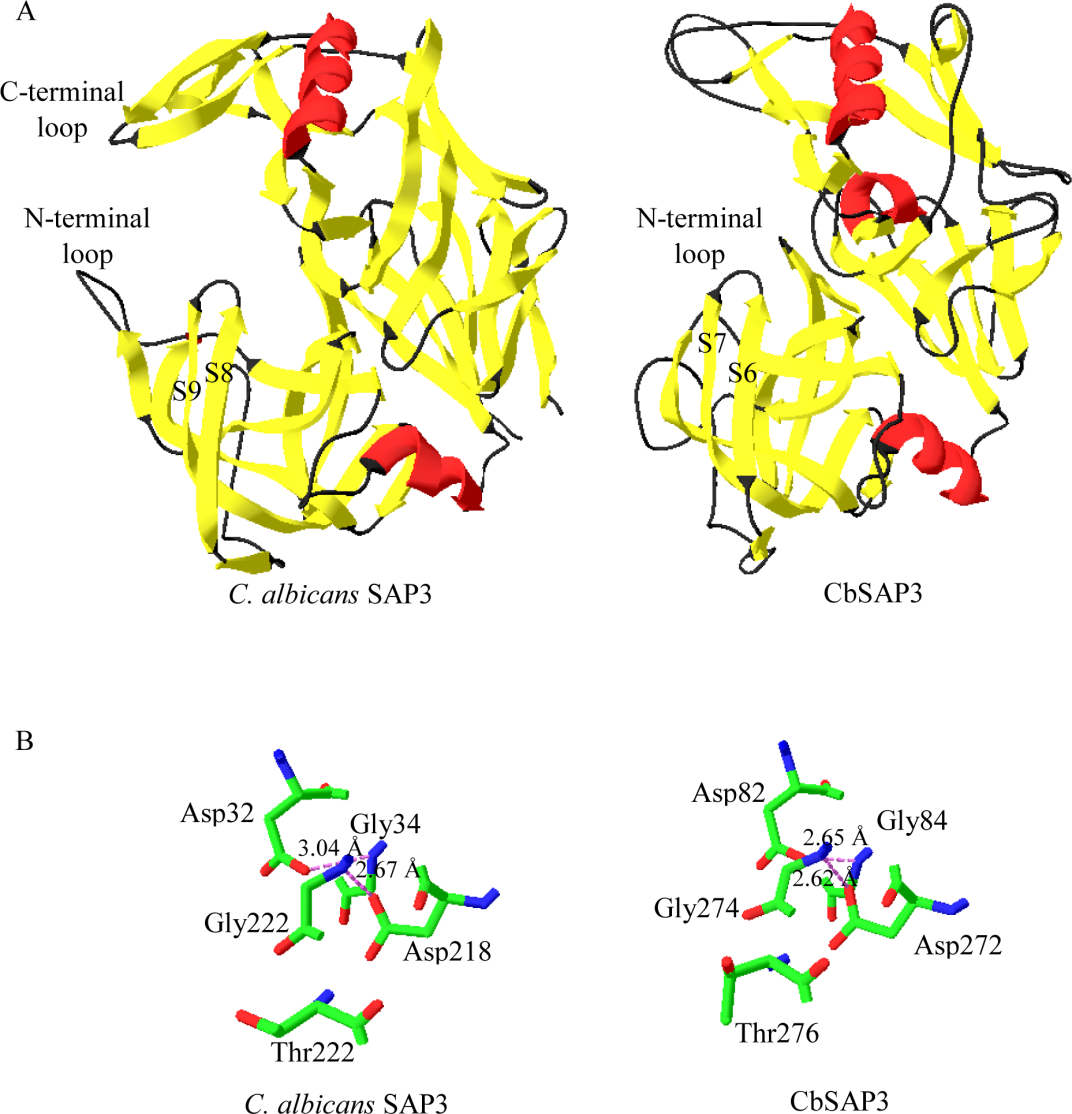
Homology model of the CbSAP3. (A) Three-dimensional ribbon structures of CbSAP3 and *C*. *albicans* SAP3. α-helices are shown in red; β-sheets are shown in yellow and random coils are shown in black. The N- and C- terminal end are labeled. (B) Active sites comparison of the CbSAP3 and *C*. *albicans* SAP3. Carbon atoms are shown in green; nitrogen atoms are shown in blue; oxygen atoms are shown in red. Hydrogen bonds are shown in pink dotted lines.

The molecular weight of the *C*. *bantiana* SAP3 (CbSAP3) is 40.71733 kDa and the isoelectric point is 3.87, computationally predicted using the ExPasy software, which are comparable to experimentally derived results [57]. The protein was mainly composed of random coils and beta sheets based on the predicted secondary structure of the CbSAP3 (S2 Fig). An approximately of 61% of the CbSAP3 structure is random coils, with 234 of its residues making up 23 coils; 29% of its structure is consisted of β-strands, with 110 of its residues making up 30 beta strands; 10% of the CbSAP3 structure is helical, with 38 of its residues making up five helices (S2 Fig). Three-dimensional (3D) homology model of CbSAP3 was generated based on the experimentally solved structural homolog using a fully automated SWISS-MODEL server. The *C*. *albicans* SAP3 (PDB: 2h6t.1.A) was used as the template for homology modeling of CbSAP3. The 40% sequence identity between CbSAP3 and *C*. *albicans* SAP3 (PDB: 2h6t.1.A) was above the 30% limit that is considered to be the threshold limit for an accurate homology modeling [58]. CbSAP3 appear as a kidney-shaped bilobed protein primarily composed of β-strands that separated into an N-terminal and a C-terminal domain (Fig 6A) which is similar to *C*. *albicans* SAP3 protein. The conserved catalytic aspartic acid active sites (Asp82 and Asp272) were located at the groove formed between the domains that is partly covered by an antiparallel β-hairpin made from β-strands 6 and 7 (121–142 residues) (Fig 6A). This β-hairpin structure is known as the active site flap in aspartyl proteinases [59, 60]. The catalytic Asp residues (Asp82 and Asp270) are located in an Asp-Thr-Gly-Ser/Tyr motif in both the N-terminal and C-terminal domains. As previously described [60], water molecule directly binds to both Asp82 and Asp272 catalytic sites surrounded by other water molecules, which interact to Gly84, Gly274, and Thr276 active site residues (Fig 6B and S3 Fig). The structures of catalytic active sites of both CbSAP3 and *C*. *albicans* SAP3 were compared (Fig 6B). In consistent with the *C*. *albicans* SAP3 structure, the conserved Asp82 residue (OD1 atom) of the CbSAP3 forms a hydrogen bond with N atom of Gly84 residue. The OD1 atom of Asp272 is hydrogen bonded to the Gly274 N atom (Fig 6B). CbSAP3 has a disulfide loop that builds up the N-terminal loop (Cys96 and Cys99) (Fig 6A). In contrast to *C*. *albicans* SAP3, no C-terminal loop was observed although a disulfide bond was predicted between Cys306 and Cys343. Apparently the most striking difference in CbSAP3 is the absence of zinc ion and pepstatin A binding sites. Accuracy of the CbSAP3 model was assessed by PROCHECK program, a protein structure validation program. The stereochemistry of main chains and side chains of the CbSAP3 model are showed in S9 Table. All the parameters of main chains and side chains were indicated to be within the acceptable limit and it can serve as a good hypothetical domain. The model could serve as a hypothetical CbSAP3 structure for further investigation of the invasive property of this pathogenic fungus.

### Secondary metabolism: melanin

Secondary metabolite production is a hallmark of filamentous fungi. These compounds are important chemicals, ranging from fatal mycotoxins to medicinal biologically active compounds that are beneficial to humankind. Secondary metabolite biosynthetic genes are frequently appeared in clusters with a main backbone gene [61]. SMURF tool was used to annotate secondary metabolite biosynthetic gene clusters and backbone genes. A total of 14 gene clusters encoding backbone enzymes were predicted in *C*. *bantiana* UM 956 genome, including five genes encoding polyketide synthase (PKS) or PKS-like, eight genes encoding non-ribosomal peptide synthetase (NRPS) or NRPS-like, and a gene encoding dimethylallyl tryptophan synthase (DMATS).

Dematiaceous fungi are remarkable microorganisms that readily produce melanins during formation of fungal spore for deposition in the cell wall. The melanins are virulence factors for many pathogenic fungi. Most fungi biosynthesized melanins through 1,8-dihydroxynaphthalene (DHN) pathway. These melanins are known as DHN-melanins. Recognized human pathogens that form DHN-melanins include *Aspergillus nidulans*, *A*. *niger*, *Alternaria alternata*, *C*. *bantiana*, *Cladosporium carrionii*, *E*. *dermatitidis*, *E*. *jeanselmei*, *Fonsecaea compacta*, *Phialophora richardsiae*, and *Neoscytalidium dimidiatum* [62]. Polyketide synthases (PKs) are important components to make DHN-melanin precursors. Here, we identified two PKS genes (UM956_2591 and UM956_7690), which is best matched to a characterized conidial yellow pigment biosynthesis PKS (WA) from *A*. *nidulans* FGSC A4 [63]. Both the PKSs contain features of a non-reducing fungal type I PKS with a starter unit of ACP transacylase (SAT), β-ketoacyl synthase (KS), acyltransferase (AT), product template (PT), acyl carrier protein (ACP), and thioesterase (TE) [64]. The PKSs have domain order of KS-AT-ACP-ACP-TE similar to the PKS that is involved in melanin biosynthesis [65]. Additionally, a set of DHN melanin-synthesis related proteins, including a scytalone dehydratase, SCD1 (UM956_4290), a tetrahydroxynaphthalene reductase, BRN1 (UM956_1427), and a transcription factor CMR1 (UM956_9060) were identified in *C*. *bantiana* UM 956. We also identified two mitogen-activated protein kinases (MAPKs), ChK1 (UM956_1033), and Mps1 (UM956_3770) that have been reported to regulate the expression of *CMR1* for pigmentation [66]. Taken together, these data revealed that *C*. *bantiana* UM 956 may produce melanin via the DHN-melanin pathway, which regulated by ChK1 and Mps1, although a gene cluster comprising *PKS*, *BRN1*, and *CMR1* was not determined in the genome.

### Iron Uptake and Homeostasis

**Fig 7:**
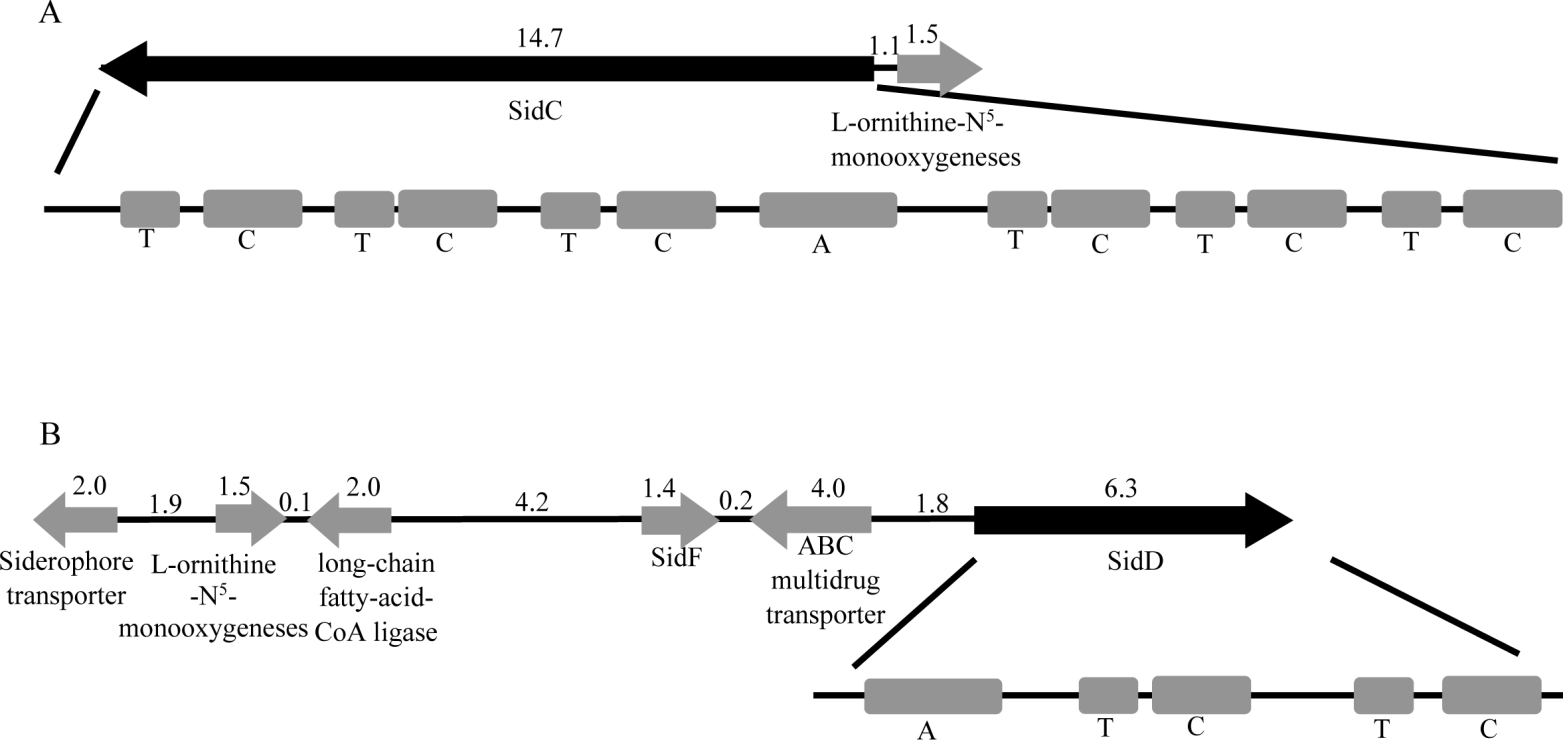
Siderophore genes of *C*. *bantiana* UM 956 genome. (A) FC synthetase *SidC* gene. (Top panel) Schematic map of *SidC* (black arrow) and adjacent genes. Gray arrows are genes which are expected to function during siderophore biosynthesis. Numbers are in kilobases. (Bottom panel) Domain setup of SidC. (B) FSC synthetase *SidD* gene. (Top panel) Schematic map of *SidD* (black arrow) and adjacent genes. Numbers are in kilobases. (Bottom panel) Domain setup of SidD.

Iron is an important biological paradox of survival factor which is indispensable for virtually all organisms including fungi. Importantly, iron acquisition represents a key step in the infection process for pathogens [67]. However, inappropriate storage of iron or iron overload can generate oxidative stress through Haber-Weiss/Fenton chemistry [68]. Thus, fungi usually employ two high-affinity iron uptake mechanisms, nonribosomally synthesized secreted iron chelators (siderophores) and non-siderophore reductive iron assimilation (RIA) [69–71]. Here, we identified two genes encoding L-ornithine-N^5^-monooxygeneses (UM956_953 and UM956_3728) that catalyze hydroxylation of L-ornithine, the first committed step for the biosynthesis of extracellular fusarinine C (FSC) and triacetylfusarinine C (TAFC), and intracellular ferricrocin (FC). Apart from SidC (UM956_3727), the NRPS crucial for FC synthesis, the FSC biosynthetic enzymes NRPS SidD (UM956_958) and SidF (UM956_956) were also identified in *C*. *bantiana* UM956. Nevertheless, the orthologue of TAFC synthetase *SidG* gene was not identified, indicating that TAFC was not synthesized by *C*. *bantiana* UM 956. SidC and SidD lack of typical domain arrangement that observed in *A*. *fumigatus* [72]. SidC has an adenylation (A), six thiolation (T), and six condensation (C) domains with the arrangement of T-C-T-C-T-C-A-T-C-T-C-T-C. The gene encoding SidC is located in close proximity to the L-ornithine-N^5^-monooxygenese (Fig 7A). SidD has a domain arrangement of A-T-C-T-C. The NRPS is located in the FSC biosynthetic gene cluster: siderophore transporter, L-ornithine-N^5^-monooxygeneses, acetyltransferase SidF, ABC multidrug transporter, and SidD (Fig 7B). Moreover, we identified a GATA-like transcription factor (UM956_2655), a homolog of URBS1 of *Ustilago maydis* [73] and of *SREA* of *A*. *nidulans* [74], that modulate siderophore biosynthesis and iron uptake. As reported in Yuan *et al*. [73], multiple GATA binding sites are present in the intergenic region between the genes encoding L-ornithine-N^5^-monooxygenese and *SidC* (S10 Table).

Alternatively, RIA is an important backup mechanism for fungi to support extracellular siderophore-driven iron homeostasis [71]. RIA is a three-step process which is regulated by metalloreductase Fre1, ferroxidase Fet3, and iron permease Ftr1. In this study, a metalloreductase Fre8 (UM956_11306), three ferroxidases Fet3 (UM956_46, UM956_979, UM956_3628), and two iron permease Ftr1 (UM956_47 and UM956_980) were identified in the genome. It should be noted that two ferroxidase and iron permease encoding gene clusters were identified. As previously reported [75, 76], the ferroxidases Fet3, UM956_46 and UM956_979 were located adjacent to iron permeases Ftr1, UM956_47 and UM956_980, respectively.

### Stress adaptation in *C*. *bantiana*

Pathogenic fungi may contain a battery of stress-responsive proteins to promote the pathogenicity and tune the physiological fitness to their diverse host niches. It is clear that *C*. *bantiana* can adapt well in animal as well as human host microenvironment. Thus, the *C*. *bantiana* UM 956 genome was mapped to the fungal stress response database (FSRD) to obtain information of stress biology of *C*. *bantiana*. A total of 311 putative proteins was assigned to their orthologous genes in the FSRD. The full list of FSRD annotation was listed in S11 Table.

*C*. *bantiana* can grow at human body temperature and up to 43°C [77]. Therefore, it is not surprising that *C*. *bantiana* contains an armory of thermal stress-responsive proteins to enable the fungus to survive in human host. *C*. *bantiana* UM 956 genome was predicted to contain HSP60 (UM956_2121), HSP70 (UM956_8246), HSP78 (UM956_6295), HSP83 (UM956_4074), HSP98 (UM956_1152), and heat shock protein ssb1 (UM956_3475) to promote degradation of damaged proteins, folding of client proteins, and to stabilize proteins and membranes [78]. Indeed, heat shock transcription factor Hsf1 is activated in response to the thermal stress and bind to the canonical heat shock elements (HSEs) in the promoters of heat shock proteins [79]. Here, an Hsf1 (UM956_7666) was identified in *C*. *bantiana* UM 956. Thermotolerance is an important virulence factor that is responsible for its pathogenicity [40]. Nicholls *et al*. [79] indicated that the activation of Hsf1 is required despite during slow thermal changes such as those suffered by febrile patients. The Hsf1-HSE regulons in *C*. *bantiana* may function to promote cellular adaptation in response to the thermal stress.

In human, phagocytic cells tend to produce an array of reactive oxygen species (ROS) such as superoxide (O^2−^) to kill fungal pathogens by damaging their deoxyribonucleic acid, proteins and membranes [80]. Thus, fungal pathogens harbor some detoxification mechanisms to respond to these chemicals. These ROS stress responses have been reported to promote the fungal pathogens’ virulence [81, 82]. In this work, we found that *C*. *bantiana* UM 956 may detoxify ROS using superoxide dismutase (SOD), catalase, and components of the thioredoxin and glutaredoxin systems to thrive against the oxidative environments created by human host. The fungus may produce two SODs: SOD2 (Mn/Fe) (UM956_1425) and SODC (Cu/Zn) (UM956_6712) that catalyze dismutation of the O^2−^ species into molecular oxygen (O^2^) or hydrogen peroxide (H_2_O_2_). *C*. *bantiana* UM 956 also contains other important genes encoding antioxidant enzymes, including seven catalases (UM956_6359, UM956_6429, UM956_7993, UM956_8082, UM956_10667, UM956_11123, and UM956_4627), thioredoxin reductase (UM956_11048), monothiol glutaredoxin, grx5 (UM956_6203), grx3 (UM956_9386), glutathione peroxidase, gpx1 (UM956_328), and methionine sulfoxide reductase (UM956_12128) were identified in the genome. Furthermore, we identified a *tpsA* gene encoding trehalose-6-phosphate synthase (UM956_10499) that involved in the first step biosynthesis of trehalose (antioxidant). Based on NCBI nr and SwissProt annotations, a series of signaling proteins and transcription factors that required for oxidative stress resistance in the fungus were determined (S12 Table). There is undoubtedly the neurotropic *C*. *bantiana* UM 956 comprises of a number of effective defense mechanisms to respond combinations of different host-generated stresses that relevant to the host-pathogen interaction.

### Antifungal susceptibility profile and drug resistance genes

According to the Epsilometer Test, the isolate is susceptible to caspofungin (MIC = 0.032 μg/mL), anidulafungin (MIC = 0.003 μg/mL), posaconazole (MIC = 0.004 μg/mL), voriconazole (MIC = 0.19 μg/mL) and itraconazole (MIC = 0.125 μg/mL). However, the isolate showed low resistance to amphotericin B (MIC = 2 μg/mL) and high resistance to fluconazole (MIC >256 μg/mL).

Compelling evidence has been gathered for the genetic basis of azole resistance development in yeast and molds. Azole resistance can be caused by mutations in the drug target enzyme, lanosterol 14α demethylase, which is encoded by the *ERG11*/*CYP51* gene or by overexpression of genes coding for membrane transport proteins [83–85]. In this study, two lanosterol 14α demethylases (UM956_1899, 97.4% and UM956_10619, 96.7%) were identified in the *C*. *bantiana* UM 956 genome. These two protein sequences were compared with the previously published ERG11 protein sequences from azole-susceptible *C*. *albicans* isolates (GenBank accession no. XM_711668 and AIX03623) to investigate the Erg11 mutations of fluconazole resistant isolate. Common ERG11 mutations (A114S, Y132F, K143R, F145L, S405F, D446E, G448E, G450E, and G464S) that related to azole resistance [86, 87] were not observed. However, nonsynonymous Erg11 mutations (D153E, E266D, and D116E) that reported in fluconazole resistant *C*. *albicans* isolates [88] were identified in UM956_1899 and UM956_10619. Single mutation (D116E) was detected in UM956_10619, while more than one nucleotide changes (D153E, E266D, and D116E) were identified in UM956_1899 (S4 Fig).

Moreover, we determined two putative NDT80 transcription factors (UM956_5350 and UM956_11916) in the genome, which are important in regulating sterol metabolism and fluconazole tolerance in *C*. *albicans* by binding to the promoters of *Erg11* gene [89]. The 500 bp upstream regions of the UM956_1899 and UM956_10619 genes were analysed *in silico* for NDT80 transcription factor binding site. Among them, only UM956_1899 gene contains a putative Ndt80 binding consensus located between −182 and −202 (S5 Fig).

Azoles resistance can be also mediated by overexpression of major facilitator superfamily (MFS) transporter encoded by *MDR1* (multidrug resistance) and ATP-binding cassette (ABC) transporters encoded by *CDR1* and *CDR2* (*Candida* drug resistance). MFS transporters contain two type of proton antiporters, including drug: H+ antiport 1 (DHA1), consisting of 12 transmembrane domains and drug: H^+^ antiport 2 (DHA2), consisting of 14 transmembrane domains [90, 91]. These efflux pumps play important role to efflux drugs in exchange with one or more H+ with a substrate molecule to confer azoles resistance in prokaryotes as well as eukaryotes [92]. In this study, a DHA1 (UM956_ 913) was identified in the *C*. *bantiana* UM 956 genome, although its expression level remain unknown. TMpred analysis predicted 12 transmembrane helices in UM956_913 (S6 Fig), suggesting it harbours transmembrane transporter activity to efflux drugs. In addition, neither CDR1 nor CDR2 was found in the *C*. *bantiana* UM 956 genome. Taken together, the ERG11, NDT80 transcription factor, and the DHA1 may work in concert to regulate ergosterol metabolism to confer fluconazole resistance in *C*. *bantiana* UM 956.

### Comparative genomics of *C*. *bantiana* and other dematiaceous fungal pathogens

**Fig 8:**
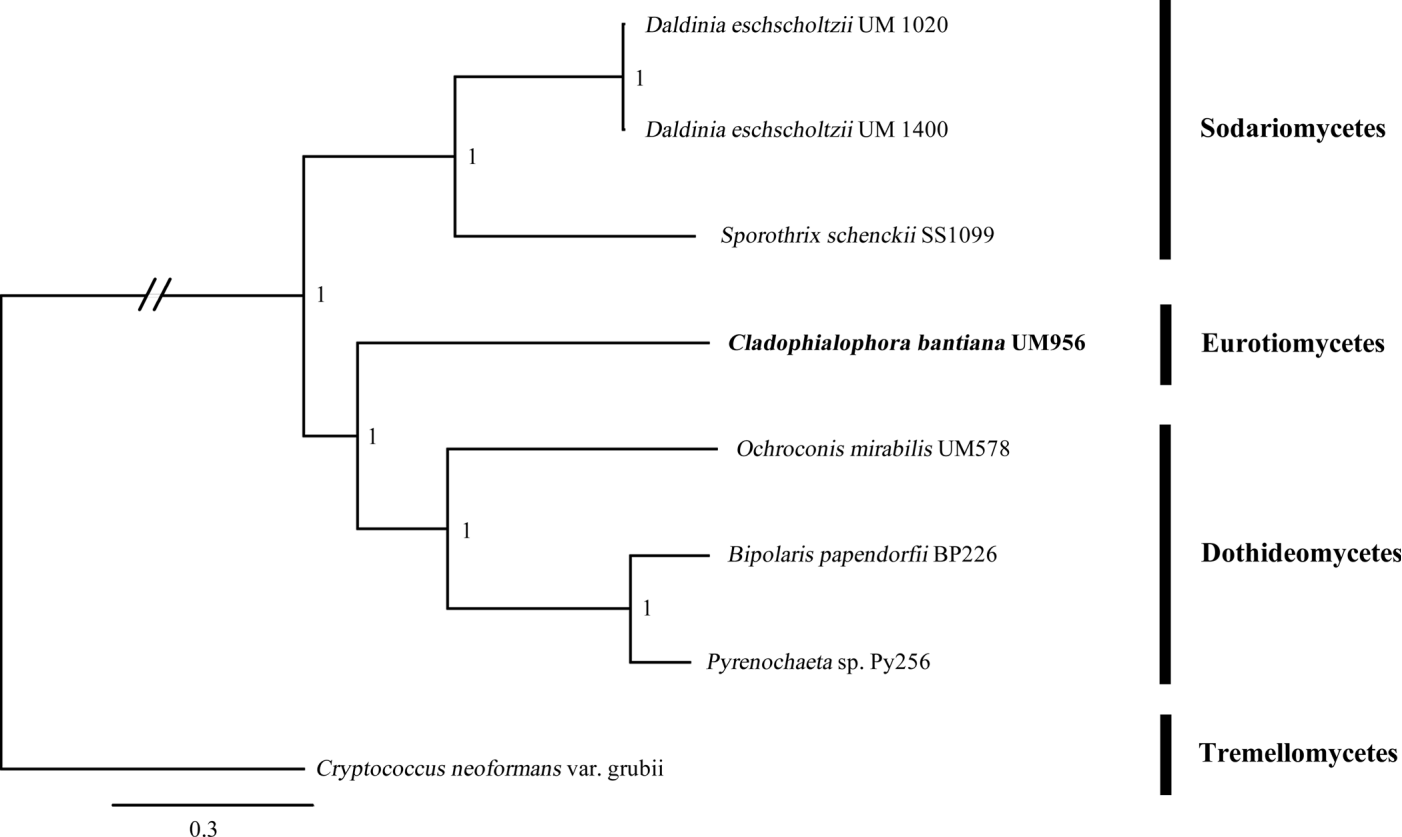
Comparative phylogenomic analysis of *C*. *bantiana* UM 956 along with eight previously published dematiaceous fungal genomes using Bayesian. Number at the node referring to Bayesian posterior probability. The tree is rooted with *C*. *neoformans var*. *grubii* H99 as outgroup.

Phylogenomic tree was constructed using nine publicly available dematiaceous fungal and a yeast pathogens from different classes, including two Eurotiomycetes, three Dothideomycetes, three Sodariomycetes and a Tremellomycetes as outgroup. A total of 98,789 proteins were clustered into 15,953 orthologous clusters with 2,199 single-copy orthologous genes determined in all the analyzed genomes. Concatenated alignments of 2,199 (13.78%) single-copy orthologous genes were used to construct Bayesian trees. From the phylogenomic trees, Sodariomycetes fungi (*D*. *eschscholzii* UM 1020, *D*. *eschscholzii* UM 1400, and *S*. *schenckii* strain 1099–18) were clustered into one clade; Dothideomycetes fungi (*O*. *mirabilis* UM 578, *Pyrenochaeta* sp. UM 256, and *B*. *papendorfii* UM 226) were grouped into a different clade, while Eurotiomycetes fungi (*Exophiala dermatitidis* NIH/UT8656 and *C*. *bantiana* UM 956) form a separate branch (Fig 8).

The core gene families between *C*. *bantiana* UM 956 and other nine genomes were identified using the OrthoMCL analysis (S13 Table). The other nine genomes used for comparison were divided into dematiaceous fungal pathogens (*B*. *papendorfii*, *D*. *eschscholtzii*, *O*. *mirabilis*, *E*. *dermatitidis*, *Pyrenochaeta* sp., and *S*. *schenckii*) and a neurotropic yeast pathogen (*C*. *neoformans var*. *grubii*) groups. Based on the orthology analysis, the gene families in *C*. *bantiana* UM 956 can be classified into three categories: i.) found in *C*. *bantiana* UM 956 and other dematiaceous fungi, 1477 gene families; ii.) found in *C*. *bantiana* UM 956 and *E*. *dermatitidis* NIH/UT8656, 921 gene families; iii.) only found in *C*. *bantiana* UM 956, 148 gene families (S14 Table). The *C*. *bantiana* UM 956 and *E*. *dermatitidis* NIH/UT8656 shared 21 family clusters with known functions (Table 5). Of these, only gene encoding calmodulin (UM956_2895) was related to fungal pathogenicity based on the pathogen-host interaction (PHI) gene analysis. Calmodulin plays a vital role in the Ca^2+^ signaling pathways. Disruption of Ca^2+/^calmodulin-dependent kinase in *Magnaporthe oryzae* not only hindered the formation of appressoria and conidial germination but also reduced its ability to infect rice plants [93]. Additionally, *C*. *bantiana* UM 956 and *E*. *dermatitidis* NIH/UT8656 shared gene families associated with stress responses, including, genes encoding myosin-cross-reactive antigen (mcra) (family Clado12877) and glucose-repressible protein, Grg-1 (family Clado13011). Myosin-cross-reactive antigen provides additional function in stress protection in bacteria [94]. Bischoff *et al*. [95] postulated that high temperature at the site of infection triggered stress in *Staphylococcus aureus* to up-regulate the expression of *mcra* gene. Fungal pathogens and saprophytes contain various glucose-repressive activities to obtain nutrients from their environments [96]. Previous studies revealed that the *grg-1* gene is regulated by glucose and its mRNA level was markedly increased when glucose was deprived [96].

**Table 5:** Shared gene families in both *C*. *bantiana* UM 956 and *E*. *dermatitidis* NIH/UT8656.

Families	Gene ID	Annotation
Clado12617	UM956_2895	Calmodulin
Clado12419	UM956_11691	Enolase
Clado13343	UM956_10094	3-oxoacyl-[acyl-carrier protein] reductase
Clado12502	UM956_1607	Hydroxymethylglutaryl-CoA lyase
Clado8358	UM956_11042	Gluconate 5-dehydrogenase
Clado12892	UM956_5601	Alpha-ketoglutarate-dependent 2,4-dichlorophenoxyacetate dioxygenase
Clado12552	UM956_2102	Glucarate dehydratase
Clado12877	UM956_5482	Myosin-cross-reactive antigen
Clado13319	UM956_1006	Serine/threonine protein kinase
Clado12819	UM956_4926	Transcription initiation factor TFIID subunit 12
Clado13293	UM956_9670	Alpha-methylacyl-CoA racemase
Clado12881	UM956_5516	myb-like DNA-binding protein Flb
Clado12590	UM956_2498	Serine/threonine protein kinase
Clado12337	UM956_10723	Regulatory protein SWI5
Clado13216	UM956_102	Alcohol dehydrogenase
Clado12541	UM956_210	Lysyl oxidase-like protein 2/3/4
Clado8464	UM956_9770	Biphenyl-2,3-diol 1,2-dioxygenase
Clado13011	UM956_6681	Glucose-repressible protein, Grg-1
Clado12706	UM956_3739	Carboxypeptidase
Clado13191	UM956_876	Glucan 1,3-beta-glucosidase
Clado13300	UM956_9747	Acetolactate synthase, catabolic

Whole proteome alignment of the *C*. *bantiana* UM 956 and the other nine fungal gene contents showed that *C*. *bantiana* UM 956 harbored 679 unique genes. Among the 148 unique gene families, only four clusters with known functions (Table 6). The predominant of the gene families were hypothetical proteins or unannotated proteins. Thus, we performed domain analysis on these unknown proteins to obtain clue of their role or function in *C*. *bantiana* UM 956. The analysis revealed that most of the hypothetical proteins related to MFS (eight clusters) and cytochrome P450 (seven clusters). Major facilitator superfamily is one of the largest families of transporters that ubiquitously found in bacteria, archaea, and eukaryotes [97]. Apart from export host-derived antimicrobial compounds as described in the previous section, MFS also involved in transport secondary metabolites, a wide array of organic and inorganic anions and cations, and essential nutrients [97]. The cytochrome P450 proteins play many roles in physiological processes, such as secondary metabolites biosynthesis, detoxification of host defense compounds, and xenobiotics degradation. In this work, we found that 14 of the unique hypothetical proteins contain cytochrome P450 domain or conserved sites. Nonetheless, the exact roles of these proteins remain to be determined. In particular, we identified two hypothetical proteins (UM956_2358 and UM956_9078) with Clr5 domain (Clado10284 family). Clr5 play a role in gene silencing in fission yeast heterochromatin by modulation of chromatin structure in the mating-type region and regulate sexual differentiation [98]. Overall, the putative unique Clr5-containing proteins might involve in the epigenetic regulation to affect gene silencing at heterochromatic loci.

**Table 6:** Specific functional family clusters in *C*. *bantiana* UM 956.

Families	Gene ID	Annotation
Clado8347	UM956_2269	Acetoacetate-CoA ligase
	UM956_7538	Acetoacetate-CoA ligase
	UM956_10163	Acetoacetate-CoA ligase
Clado12590	UM956_2498	Serine/threonine protein kinase
Clado13242	UM956_9168	Serine/threonine protein kinase
Clado10301	UM956_4621	Succinate-semialdehyde dehydrogenase (NADP+)
	UM956_9931	Succinate-semialdehyde dehydrogenase (NADP+)

## Conclusions

The report represents the first case of brain abscess caused by *C*. *bantiana* in Malaysia and Southeast Asia. *C*. *bantiana* UM 956 was identified using combined morphological examination and multigene phylogeny. The infected patient was successfully treated with a combination of surgical excision and antifungal therapy. Excision rather than aspiration combined with systemic long term antifungal therapy is essential for resolution of fungal cerebral phaeohyphomycosis infection. In this study, we successfully produced a high-quality draft genome sequence of *C*. *bantiana* UM 956. The *C*. *bantiana* UM 956 constitutes a repertoire of genes for the saprophytic lifestyle as well as invasion action of the pathogen. Moreover, our genome analysis revealed that the neurotrophic fungus can respond to multiple environmental stresses to enable it to survive in human host.

## Materials and Methods

### Ethic statement

Approval for this study was obtained from the Medical Research and Ethic Committee (MREC), Ministry of Health (MOH) (reference number: (7) KKM/NIHSEC/P151131), and written consent was obtained.

### Fungal isolate

*C*. *bantiana* UM 956 was recovered from the brain tissue of a patient with brain abscess in the Mycology Unit, Department of Medical Microbiology, UMMC, Kuala Lumpur. The isolate was processed according to the laboratory’s standard operating procedures (SOP) with direct wet mount microscopy followed by inoculation on SDA for incubation at 30°C up to seven days, with alternate day examination for fungal growth. Macroscopic examination was performed to observe its colonial morphology, such as color, texture, and topography. Tease mount and slide culture were performed to identify the arrangement of conidia and conidiophores.

### *In vitro* antifungal susceptibility

The *in vitro* antifungal susceptibility of the fungal isolate was evaluated by the Epsilometer Test (Etest, Biomerieux, France) by determining the minimum inhibitory concentrations (MICs) according to the previous study [99]. The isolate was tested against nine antifungal drugs, including fluconazole, itraconazole, posaconazole, voriconazole, anidulafungin, caspofungin, micafungin, 5-flucytosine, and amphotericin B.

### DNA sequencing and phylogenetic analysis

The internal transcribed spacer region (ITS), the small subunit of the ribosomal RNA gene (SSU), and the large subunit of the ribosomal RNA gene (LSU) were used as targets for molecular identification of the clinical isolate. Total DNA extraction, PCR amplification, sequencing, and BLASTn search were performed as described previously [26, 99]. Unique ITS and LSU sequences from the isolate, together with an additional 12 species of *Cladophialophora* and an outgroup strain of *P*. *schaereri* (Table 1) were subjected to phylogenetic analysis. Multiple sequence alignments of collected ITS and D1/D2 nucleotide sequences were generated using M-Coffee [100]. Individual alignments were concatenated for Bayesian Markov Chain Monte Carlo (MCMC) analysis partitioned by the gene. Bayesian tree analyses were performed using MrBayes v3.2.2 with reversible jump MCMC averaging over the entire general time reversible (GTR) rates and gamma-distributed rate heterogeneity for all subsets of the partitioned scheme. A total of 500,000 generations were run with a sampling frequency of 100, and diagnostics were calculated for every 1,000 generations. The first 1,250 trees were discarded with a burn-in setting of 25%. Convergence was assessed with a standard deviation of split frequencies below 0.01, no noticeable trend for the plot of the generation versus the log probability of the data, and a potential scale reduction factor (PSRF) close to 1.0 for all parameters.

### Genomic DNA extraction, genome sequencing and assembly

Genomic DNA of *C*. *bantiana* UM 956 was extracted as described previously [26, 34]. The genome was sequenced using Illumina HiSeq 2000 Sequencer (Illumina Inc., San Diego, CA USA) in a 2×90 bp paired-end mode on 500-bp and 5-kb library sizes. Illumina library was prepared using TruSeq v3 Reagent Kits (Illumina Inc., San Diego, CA USA). The selected library fragments were purified through gel electrophoresis, which then selectively enriched and amplified by PCR. The 500-bp Illumina sequenced read was then combined with the 5-kb Illumina sequenced read for further processing. Both sets of sequenced reads were first pre-processed using FASTX-Toolkit (http://hannonlab.cshl.edu/fastx_toolkit/) trimming bases with a Phred quality below Qv20 from the 3’-end of the reads. The trimmed reads shorter than 30 bp and reads with 40% bases having Qv ≤ 20 were filtered out, retaining small-insert reads ≥ 80 bp and large-insert reads ≥ 30 bp. Two bases were trimmed from the 5’-terminal of all reads. Pre-processed reads from both libraries were assembled with Velvet version 1.2.07 [101] with k-mer setting = 73, insert length = 568, -ins_length_sd = 100, -min_pair_count = 15, insert_length2 = 5000, ins_length2_sd = 500, and min_contig_lgth = 200. Additional parameter of -shortMatePaired = yes was set for large insert library. The generated contig assembled from the Velvet were further scaffolded using SSPACE Basic v2.0 [102] with more stringent parameters than software default to achieve higher accuracy assembly (parameters: -z 200, -k 15, -a 0.3, -n 30 and -T 10). GapFiller v1.10 (-m = 60, -o = 15, -r = 0.8,–n = 30, -t = 30 and -T = 10) was used to perform gap filling by utilizing paired-end sequencing data from both libraries [103, 104].

### Genome annotation of *C*. *bantiana* UM 956

Interspersed repetitive elements and low complexity DNA sequences were masked using RepeatMasker version open-3.3.0 with the Repbase fungal library version rm-20120418, followed by masking off the RNA sequences. GeneMark-ES version 2.3e [105] was used for gene prediction in *C*. *bantiana* UM 956 complete genome. Annotation of coding sequences for UM 956 was completed using BLAST (Basic Local Alignment Search Tool) searches against the NCBI nr protein and SwissProt databases. Protein domain families were matched to Pfam database using InterProScan 5 [106]. Individual rRNA and tRNA were predicted using RNAmmer v1.2 [107] and tRNAscan-SE v1.3.1 [108], respectively. Putative transposable elements were identified using Transposon-PSI (http://transposonpsi.sourceforge.net) by PSI-TBLASTN searches with a collection of (retro-) transposon open reading frame (ORF) homology profiles.

### Functional annotation of predicted genes

Kyoto Encyclopedia of Genes and Genomes (KEGG) metabolic pathways matches was carried out using local BLAST2GO tools [109]. Protein classification was performed using EuKaryotic Orthologous Group (KOG) [110]. The total number of predicted proteins involved in the KOG category “Secondary metabolites biosynthesis, transport and catabolism” for other pathogenic dematiaceous fungi was obtained from the DemaDB database (fungaldb.um.edu.my) [111].

The best protein models of the *C*. *bantiana* UM 956 genome was subjected to database of automated Carbohydrate-active enzyme ANnotation (dbCAN) annotation pipeline [112]. Comparative analysis was performed against fungi with different lifestyles, including saprophytic fungi (*Neurospora crassa* OR74A and *Trichoderma reesei* QM6a), facultative parasitic fungi (*A*. *nidulans* FGSC A4), biotrophic fungi (*C*. *flavum*, *Ustilago maydis*, and *Blumeria graminis*), necrotrophic fungi (*Cochliobolus heterostrophus* C4 and *C*. *heterostrophus* C5), hemi-biotrophic fungi (*Cochliobolus sativus* ND90Pr, *Magnaporthe oryzae* 70–15 version 8, and *Fusarium graminearum* PH-1), and symbiotic fungus (*Laccaria bicolor*) [39, 113].

Peptidases was identified by a batch blast of *C*. *bantiana* UM 956 protein models against MEROPS database [114]. Secreted peptidases were determined by the cleavage sites prediction and signal peptide/non-signal peptide using SignalP version 4.12 [115] after discarding proteins with transmembrane domains that were determined by TMHMM version 2.0 [116]. Secreted peptidases were selected based on the proteins without transmembrane domain or the presence of a transmembrane domain located at in the N-terminal 40 amino acids as it is responsible to the secretion signal.

Genome mapping of secondary metabolite backbone genes and associated genes for secondary metabolite biosynthesis cluster were carried out using web-based SMURF (Secondary Metabolite Unknown Regions Finder) (www.jcvi.org/smurf/) [117]. The organisation SidC and SidD gene clusters were retrieved from the sequenced genome using Artemis v12.0 sequence viewer [118]. Stress responsive genes and pathogenicity-associated genes were predicted by BLASTP search against fungal stress response database (FSRD) and pathogen-host interaction database (PHI-base). Amino acid sequences with e-value threshold ≤1e-5, alignment length over 70% of its own length and over 50% match identity were assigned as the annotation of putative genes.

### Orthologous genes and comparative genomic analysis

The protein sequences of all current publicly available dematiaceous fungal genomes (*B*. *papendorfii* UM 226 [26], *D*. *eschscholtzii* UM 1020 and UM 1400 [27], *O*. *mirabilis* UM 578 [28], *Pyrenochaeta* sp. UM 256 [29], *S*. *schenckii* strain 1099–18 [30], and *E*. *dermatitidis* NIH/UT8656 [31]) and a neurotrophic yeast genome (*C*. *neoformans var*. *grubii* H99 [17]) were used to determine the orthologous genes in *C*. *bantiana* UM 956. The genome sequences of *B*. *papendorfii* UM 226, *D*. *eschscholtzii* UM 1020 and UM 1400, *O*. *mirabilis* UM 578, and *Pyrenochaeta* sp. UM 256 were downloaded from DemaDb database (fungaldb.um.edu.my) [111]; the *S*. *schenckii* strain 1099–18 genome sequence was acquired from Laboratorio Nacional de Computacao Cientifica; the *C*. *neoformans var*. *grubii* H99 genome sequence was obtained from Broad Institute. The OrthoMCL version 2.02 [119] was used in the analysis of protein sequences clustering (≥33 amino acids) for *C*. *bantiana* UM 956 and the nine genome references by all-against-all BLASTp searches of all proteins. The reciprocal best hits from distinct genomes were identified as orthologous genes.

### Phylogenomic analysis

A phylogenomic tree was made using all proteome clusters of the seven dematiaceous fungal species produced from comparative analysis. The tree is rooted with *C*. *neoformans var*. *grubii* H99 as outgroup. A total of 2,272 single-copy orthologous genes containing one member in each species was subjected to individual sequence alignments using ClustalW version 2.0 [120]. TrimAL (with–gt 0.5) was used to discard all spurious sequences or poorly aligned regions. The filtered multiple alignments were then concatenated into a superalignment with 1,239,944 characters. Bayesian phylogenetic analysis was run using MrBayes v3.2.2 [121] with mixed amino acid model, gamma-distributed rate variation across sites, and a proportion of invariable sites. The MCMC was run using a sampling frequency of 100 for 100,000 generations with a burn-in setting of 25%

### Prediction of secondary structure and homology modelling

Secondary structure of the CbSAP3 was predicted using PSIPRED v3.3 [122]. Amino acid sequence alignment between CbSAP3 and *C*. *albicans* SAP3 was performed using Clustal Omega. Three-dimensional structural models of the CbSAP3 was produced using SWISS-MODEL server [123] using the template (*C*. *albicans* SAP3, Protein Data Bank entry 2h6t.1.A) [60] selected from template identification function in the SWISS-MODEL program. The generated model was stored as a PDB output file and the predicted structure and *C*. *albicans* SAP3 protein were visualized and compared using Swiss-PdbViewer 4.0.1. The quality of the protein model generated was validated using the PROCHECK program as described in Kuan *et al*. [124].

### Identification of GATA-like and NDT80 transcription factors binding elements

The intergenic region between the genes encoding L-ornithine-N^5^-monooxygenese and SidC and the 500 bp of upstream regions of *Erg11* genes were retrieved from the *C*. *bantiana* UM 956 genome using Artemis v12.0 sequence viewer. Consensus GATA-like and NDT80 transcription factor binding elements were predicted using the JASPAR database (http://jaspar.genereg.net/cgi-bin/jaspar_db.pl).

### Nucleotide sequence accession numbers

The ITS, SSU, and LSU nucleotide sequences of *C*. *bantiana* UM 956 were deposited in GenBank with accession numbers KU928131, KU928132, and KU928133, respectively. The assembly of the *C*. *bantiana* UM 956 was deposited in European Nucleotide Archive with BioProject number PRJEB13102 (sequence accession: FLVJ01000001-FLVJ01000426).

## Supporting Information

S1 Fig Cranial CT scan showing a large, irregular rim-enhancing necrotic lesion in the left parietal region close to the motor strip causing significant mass effect with surrounding edema. (PDF)

S2 Fig Secondary structure prediction of the CbSAP3. (PDF)

S3 Fig Amino acid sequence alignment between CbSAP3 and *C*. *albicans* SAP3. Sequence alignment was performed using Clustal Omega. Asterisk (*) indicates positions of conserved catalytic active sites.
(PDF)

S4 Fig Amino acid sequence alignment of *C*. *bantiana* Erg11 (UM956_1899 and UM956_10619) and *C*. *albicans* Erg11 (XM_711668 and AIX03623). Sequence alignment was performed using Clustal Omega. Asterisk (*) indicates positions of point mutations that related to azole resistance.
(PDF)

S5 Fig Sequence analysis of *Erg11*’-flanking region. Underlined sequences are the Ndt80 transcription factor binding sites predicted using the JASPAR database. Translation start codon ATG is indicated with boldface and boxed.
(PDF)

S6 Fig Transmembrane domains of DHA1 (UM956_ 913) as predicted by TMpred. The horizontal line represents the level of hydrophobicity (score ≥ 500) that predicts membrane-spanning domains with high probability. Predicted transmembrane domains are indicated with Roman numerals. The X axis is the amino acid sequences of the enzyme and the Y axis is the hydrophobicity of the residues.
(PDF)

S1 Table Basic genome statistics of *C*. *bantiana* UM 956 and other previously sequenced fungal genomes. (XLSX)

S2 Table List of protein-coding genes identified in *C*. *bantiana* genome based on the NCBI nr, SwissProt, and InterPro databases. (XLSX)

S3 Table List of hypothetical proteins predicted in *C*. *bantiana* UM 956 genome. (XLSX)

S4 Table KOG classification of predicted proteins in *C*. *bantiana* UM 956 genome. (XLT)

S5 Table Distribution of predicted proteins from *C*. *bantiana* UM 956 genome that involved in in KEGG metabolic pathway. (XLSX)

S6 Table CAZyme class annotation distribution in *C*. *bantiana* UM 956 genome. (XLS)

S7 Table Plant cell wall degrading and modifying CAZyme families predicted in *C*. *bantiana* UM 956 genome. (XLS)

S8 Table List of genes encoding peptidases predicted using MEROPS analysis. (XLS)

S9 Table Stereochemistry of the CbSAP3 protein model validated using PROCHECK. (XLS)

S10 Table Predicted GATA binding sites in the intergenic region between the genes encoding L-ornithine-N5-monooxygenese and SidC. (XLSX)

S11 Table List of predicted genes encoding stress response proteins identified based on FSRD. (XLS)

S12 Table List of signaling proteins and transcription factors that involved in adaptive responses to osmotic stress. (XLS)

S13 Table List of gene families and the distribution of the genes in each families between *C*. *bantiana* UM 956 and other tested fungi/yeast. BP226: *B*. *papendorfii* UM 226; Cl956: *C*. *bantiana* UM 956; CN99: *C*. *neoformans var*. *grubii* H99; DE1020: *D*. *eschscholtzii* UM 1020; DE1400: *D*. *eschscholtzii* UM 1400; OM578: *O*. *mirabilis* UM 578; Py256: *Pyrenochaeta* sp. UM 256 and SS1099: *S*. *schenckii* strain 1099–18; ED: *E*. *dermatitidis* NIH/UT8656.
(XLSM)

S14 Table Gene families shared between *C*. *bantiana* UM 956 and other tested fungi/yeast. BP226: *B*. *papendorfii* UM 226; Cl956: *C*. *bantiana* UM 956; CN99: *C*. *neoformans var*. *grubii* H99; DE1020: *D*. *eschscholtzii* UM 1020; DE1400: *D*. *eschscholtzii* UM 1400; OM578: *O*. *mirabilis* UM 578; Py256: *Pyrenochaeta* sp. UM 256 and SS1099: *S*. *schenckii* strain 1099–18; ED: *E*. *dermatitidis* NIH/UT8656.
(XLSX)
