# Supplementary figures and images for: Genomic Analyses of Cladophialophora bantiana, a Major Cause of Cerebral Phaeohyphomycosis Provides Insight into Its Lifestyle, Virulence and Adaption in Host
Source: PLoS One. 2016 Aug 29;11(8):e0161008. doi: 10.1371/journal.pone.0161008 (PMC5003357; doi:10.1371/journal.pone.0161008)

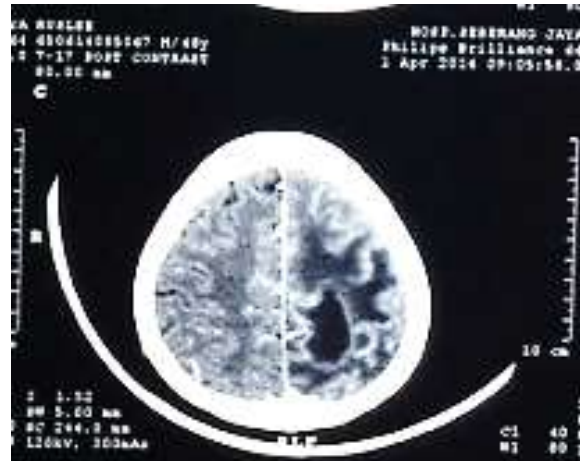

Supplement: S1 Fig — (PDF) [file pone.0161008.s001.pdf]

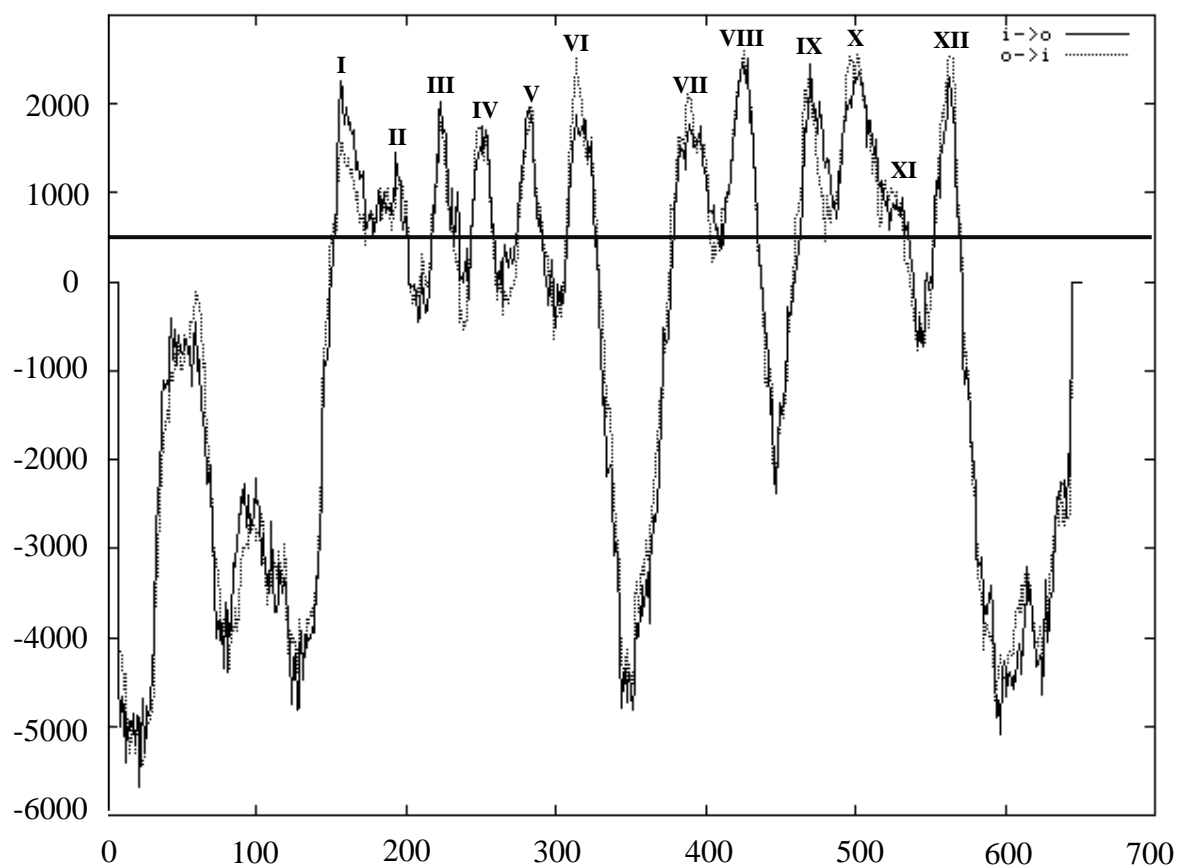

Supplement: S6 Fig — The horizontal line represents the level of hydrophobicity (score ≥ 500) that predicts membrane-spanning domains with high probability. Predicted transmembrane domains are indicated with Roman numerals. The X axis is the amino acid sequences of the enzyme and the Y axis is the hydrophobicity of the residues. (PDF) [file pone.0161008.s006.pdf]
