# Supplementary material for: Genomic Analyses of Cladophialophora bantiana, a Major Cause of Cerebral Phaeohyphomycosis Provides Insight into Its Lifestyle, Virulence and Adaption in Host
Source: PLoS One. 2016 Aug 29;11(8):e0161008. doi: 10.1371/journal.pone.0161008 (PMC5003357; doi:10.1371/journal.pone.0161008)
Supplement: S2 Fig — (PDF) [file pone.0161008.s002.pdf]

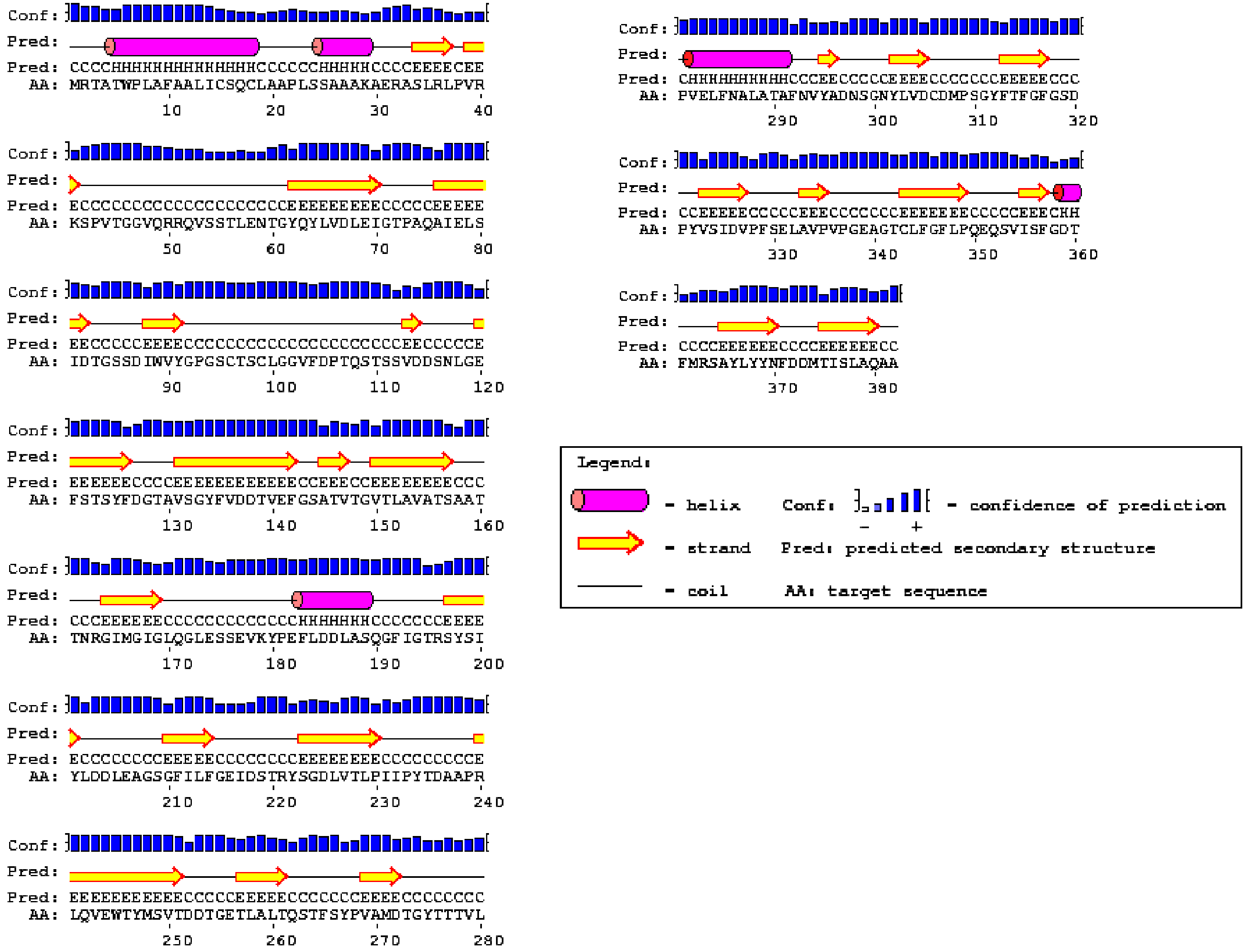
Conf:
Pred:
Pred: CCCCHHHHHHHHHHHHHHCCCCCHHHHHHCCCCEEEECEE
AA: MRTATWPLAFAALICSQCLAAPLSSAAAKAERASLRLPVR
10 20 30 40
Conf:
Pred:
Pred: ECCCCCCCCCCCCCCCCCCCCEEEEEEEEECCCCCEEEEE
AA: KSPVTGGVQRRQVSSTLENTGYQYLVDLEIGTPAQAIELS
50 60 70 80
Conf:
Pred:
Pred: EECCCCCEEEECCCCCCCCCCCCCCCCCCCCEECCCCCCE
AA: IDTGSSDIWVYGPGSCTSCLGGVFDPTQSTSSVDDSNLGE
90 100 110 120
Conf:
Pred:
Pred: EEEEEECCCCEEEEEEEEEEECCEEECCEEEEEEEEECCC
AA: FSTSYFDGTAVSGYFVDDTVEFGSATVTGVTLAVATSAAT
130 140 150 160
Conf:
Pred:
Pred: CCCEEEEEEECCCCCCCCCCCHHHHHHHCCCCCCCEEEEE
AA: TNRGIMGIGLQGLESSEVKYPEFLDDLASQGFIGTRSYSI
170 180 190 200
Conf:
Pred:
Pred: ECCCCCCCCEEEEECCCCCCCCEEEEEEEECCCCCCCCCE
AA: YLDDLEAGSGFILFGEIDSTRYSGDLVTLPIIPYTDAAPR
210 220 230 240
Conf:
Pred:
Pred: EEEEEEEEEEECCCCCEEEEECCCCCCCEEEECCCCCCCC
AA: LQVEWTYMSVTDDTGETLALTQSTFSYPVAMDTGYTTTVL
250 260 270 280
Conf:
Pred:
Pred: CHHHHHHHHHHCCCEECCCCCEEEECCCCCCCEEEEECCC
AA: PVELFNALATAFNVYADNSGNYLVDCDMPSGYFTFGFGSD
290 300 310 320
Conf:
Pred:
Pred: CCEEEEECCCCCEEECCCCCCCEEEEEEECCCCCEEECHH
AA: PYVSIDVPFSELAVPVPGEAGTCLFGFLPQEQSVISFGDT
330 340 350 360
Conf:
Pred:
Pred: CCCCEEEEEECCCCEEEEEECC
AA: FMRSAYLYYNFDDMTISLAQAA
370 380
Legend:
- helix
Conf:
- confidence of prediction
−
+
- strand
Pred: predicted secondary structure
- coil
AA: target sequence

1 M R T A T W P L A F A A L I C S Q C L A A P L S S A A A K A E R A S L R L P V R K S P V T G G V Q R 50
51 R Q V S S T L E N T G Y Q Y L V D L E I G T P A Q A I E L S I D T G S S D I W V Y G P G S C T S C L 100
101 G G V F D P T Q S T S S V D D S N L G E F S T S Y F D G T A V S G Y F V D D T V E F G S A T V T G V 150
151 T L A V A T S A A T T N R G I M G I G L Q G L E S S E V K Y P E F L D D L A S Q G F I G T R S Y S I 200
201 Y L D D L E A G S G F I L F G E I D S T R Y S G D L V T L P I I P Y T D A A P R L Q V E W T Y M S V 250
251 T D D T G E T L A L T Q S T F S Y P V A M D T G Y T T T V L P V E L F N A L A T A F N V Y A D N S G 300
301

KEY	Helix	Sheet	Disordered	Disordered protein binding	Dompred Boundary	DomSSEA Boundary
Annotations	H	L				
