# Supplementary material for: Genomic Analyses of Cladophialophora bantiana, a Major Cause of Cerebral Phaeohyphomycosis Provides Insight into Its Lifestyle, Virulence and Adaption in Host
Source: PLoS One. 2016 Aug 29;11(8):e0161008. doi: 10.1371/journal.pone.0161008 (PMC5003357; doi:10.1371/journal.pone.0161008)
Supplement: S3 Fig — Sequence alignment was performed using Clustal Omega. Asterisk (*) indicates positions of conserved catalytic active sites. (PDF) [file pone.0161008.s003.pdf]

```
                                             *   *
CbSAP3                 1 MRTATWPLAFAALICSQCLAAPLSSAAAKAERASLRLPVRKSPVTGGVQRRQVSSTLENT
C. albicans SAP3       1 --------------------------------------------------QTVPVKLINE

                                              * *
CbSAP3                61 GYQYLVDLEIGTPAQAIELSIDTGSSDIWVYGPGSCTSCLGG------------VFDPTQ
C. albicans SAP3      11 QVSYASDITVGSNKQKLTVVIDTGSSDLWVPDSQ--VSCQAGQGQDPNFCKNEGTYSPSS

CbSAP3               109 STSSVDDSNLGEFSTSYFDGTAVSGYFVDDTVEFGSATVTGVTLAVATSAATTNRGIMGI
C. albicans SAP3      69 SS--SSQNLNSPFSIEYGDGTTSQGTWYKDTIGFGGISITKQQFADVT-STSVDQGILGI

CbSAP3               169 GLQGLESSEVKYPEFLDDLASQGFIGTRSYSIYLDDLEAGSGFILFGEIDSTRYSGDLVT
C. albicans SAP3     126 GYKTHE-AEGNYDNVPVTLKNQGIISKNAYSLYLNSRQATSGQIIFGGVDNAKYSGTLIA

                                                                    * * *
CbSAP3               229 LPIIPYTDAAPRLQVEWTYMSVTDDTGETLALTQSTFSYPVAMDTGYTTTVLPVELFNAL
C. albicans SAP3     185 LPVTSDNELRIHLNT----VKVA---GQSI-----NADVDVLLDSGTTITYLQQGVADQV

UM_10742  CbSAP3     289 ATAFN----VYADNSGNYLVDCDMPSGYFTFGFGSDPYVSIDVPFSELAVPVPGEAGTCL
C. albicans SAP3     233 ISAFNGQETYDANGNLFYLVDCNLSG-SVDFAF--DKNAKISVPASEFTAPLYTEDGQVY

CbSAP3               345 F--GFLPQEQSVISFGDTFMRSAYLYYNFDDMTISLAQAA-----------
C. albicans SAP3     290 DQCQLLFGTSDYNILGDNFLRSAYIVYDLDDNEISLAQVKYTTASNIAALT
```
