# Supplementary material for: Genomic Analyses of Cladophialophora bantiana, a Major Cause of Cerebral Phaeohyphomycosis Provides Insight into Its Lifestyle, Virulence and Adaption in Host
Source: PLoS One. 2016 Aug 29;11(8):e0161008. doi: 10.1371/journal.pone.0161008 (PMC5003357; doi:10.1371/journal.pone.0161008)
Supplement: S4 Fig — Sequence alignment was performed using Clustal Omega. Asterisk (*) indicates positions of point mutations that related to azole resistance. (PDF) [file pone.0161008.s004.pdf]

```
XM_711668        1 MAIVETVIDGINY-FLSLSVTQQISILLGV--PFVYNLVWQ---YLYSLRKDRAPLVFYW
AIX03623         1 MAIVETVIDGINY-FLSLSVTQQISILLGV--PFVYNLVWQ---YLYSLRKDRAPLVFYW
UM956_1899       1 MGLLAALSKPAERLLVSLPTWQ--LVLLGFTAFLVLCVAWNVLRQVLFKDKDAPPEVWSW
UM956_10619      1 ----------MSTLIV--------KILTSLVWVIVISILTNILKQLFFRNPHRPPLVWHW

XM_711668       55 IPWFGSAASYGQQPYEFFESCRQKYGDVFSFMLLGKIMTVYLGPKGHEFVFNAKLSDVSA
AIX03623        55 IPWFGSAASYGQQPYEFFESCRQKYGDVFSFMLLGKIMTVYLGPKGHEFVFNAKLSDVSA
UM956_1899      59 FPVVGNTIWYGMDPFDFFFTCKEKYGDVFTFILLGRRVTVCLGTKGNEFILNGKLKDVNA
UM956_10619     43 FPLIGSTVDYGQDPYRFFFKCREKYGDFFTFGLLGMKVTVFLGAKGNNFILNGKLKDLNA

                    *                                    *
XM_711668      115 EDAYKHLTTPVFGKGVIYDCPNSRLMEQKKFAKFALTTDSFKRYVPKIREEILNYFVTDE
AIX03623       115 EDAYKHLTTPVFGKGVIYDCPNSRLMEQKKFAKFALTTDSFKRYVPKIREEILNYFVTDE
UM956_1899     119 EEIYTGLTTPVFGEGVVYDCPNSKLMEQKKFVKFGLTMEAFQSYVDLISSETRSFVEKGK
UM956_10619    103 EEVYGPLTVPVFGRGVVYDVDNARFMDQKRLLKEGFTSQNLRAYVPQFVKETEQYINTNA

XM_711668      175 SFKLKEKTHGVANVMKTQPEITIFTASRSLFGDEMRRIFDRSFAQLYSDLDKGFTPINFV
AIX03623       175 SFKLKEKTHGVANVMKTQPEITIFTASRSLFGDEMRRIFDRSFAQLYSDLDKGFTPINFV
UM956_1899     179 LFQA---HSGKIDVVPAMAELTIYTASRSLQGPEVRAKFDSTFADLYHDLDMGFSPINFM
UM956_10619    163 AFRG---EGGVCDISTVLSEISLYAAAGSLQGKEVRNSFDSSFATYYRHLDDGFAPVNFM

                                                  *
XM_711668      235 FPNLPLPHYWRRDAAQKKISATYMKEIKSRRERGDIDPNRD-LIDSLLIHSTYKDGVKMT
AIX03623       235 FPNLPLPHYWRRDAAQKKISATYMKEIKSRRERGDIDPNRD-LIDSLLIHSTYKDGVKMT
UM956_1899     236 LSWAPLPHNRKRDIAQKKMTDTYMEIIKQRRDAGGKREKGDEDMIWNLMGCVYKDGTPVP
UM956_10619    220 FPWLPIPVNRRRDRAQKMMANLYMDIIKKRRSNGNQD--DSHDMLWALMDGRYKDGTRLS

XM_711668      294 DQEIANLLIGILMGGQHTSASTSAWFLLHLGEKPHLQDVIYQEVVELLKEKGGDLNDLTY
AIX03623       294 DQEIANLLIGILMGGQHTSASTSAWFLLHLGEKPHLQDVIYQEVVELLKEKGGDLNDLTY
UM956_1899     296 DREVAHMMIALLMAGQHSSSSTSSWILLRLATRPDIQEDLLEEQKRV---LGEDLPPLTY
UM956_10619    278 DEEIANLMIALLMGGQHNTAASGTWIMLHLAHRPQLIQELYQEQLNV---LGGQAP--TY

XM_711668      354 EDLQKLPSVNNTIKETLRMHMPLHSIFRKVTNPLRIPET------NYIVPKGHYVLVSPG
AIX03623       354 EDLQKLPSVNNTIKETLRMHMPLHSIFRKVTNPLRIPET------NYIVPKGHYVLVSPG
UM956_1899     353 ENLQKLPLNAQVVKETLRLHAPIHSILRKVKSPMTIVANTPTTTKTYQIPTTHTLLSAPG
UM956_10619    333 DTLQDLTLHNNVIKETLRLHSPIHSIMRKVKQPMQIPET------DVVVPAGHILLAAPG

XM_711668      408 YAHTSERYFDNPEDFDPTRWDTAAAKANS------VSFNSSDEVDYGFGKVS-KGVSSPY
AIX03623       408 YAHTSERYFDNPEDFDPTRWDTAAAKANS------VSFNSSDEVDYEFGKVS-KGVSSPY
UM956_1899     413 VTSRESEFFPDPMTWEPHRWDEGHPLAYTRMG-----MDKEEFEDYGYGMIS-KGASSPY
UM956_10619    387 VPSRCEEFFPDPMAWKPHRWDKPDEAPKQQQQQQGEKGEDNDTIDYGYGAVSSKAVNSPY

XM_711668      461 LPFGGGRHRCIGEQFAYVQLGTILTTFVYNLRWTIDG--YKVPDPDYSSMVVLPTEPAEI
AIX03623       461 LPFGGGRHRCIGEQFAYVQLGTILTTFVYNLRWTIDG--YKVPDPDYSSMVVLPTEPAEI
UM956_1899     467 LPFGAGRHRCIGEQFAYVQLGAVLATIVRLVKFRQIEGKELV-PTDYSSLFSRPMAPAVV
UM956_10619    447 LPFGAGRHRCVGETFAYAQLGAILATMVRLLQWEQVDPNAPVPATDYSSMFSRPMHPATI

XM_711668      519 IWEKRETCMF
AIX03623       519 IWEKRETCMF
UM956_1899     526 EFEKREKV--
UM956_10619    507 KWRRRH----
```
