# Supplementary material for: Genomic Analyses of Cladophialophora bantiana, a Major Cause of Cerebral Phaeohyphomycosis Provides Insight into Its Lifestyle, Virulence and Adaption in Host
Source: PLoS One. 2016 Aug 29;11(8):e0161008. doi: 10.1371/journal.pone.0161008 (PMC5003357; doi:10.1371/journal.pone.0161008)
Supplement: S5 Fig — Underlined sequences are the Ndt80 transcription factor binding sites predicted using the JASPAR database. Translation start codon ATG is indicated with boldface and boxed. (PDF) [file pone.0161008.s005.pdf]

NDT80

agtccatcaattgtccaaattaattttctggcggccacttgctaaatagtcggctgcgct-160

gccaccaacaactttaccatactccccgtcaattcacgacaaccacgttcgttccctccc-100

tctgtcgttccgcctctgtcgttccgcctctgtcttccatcaccctcacttgacctctat-40

tcctagcgcaccgccgtttcccgcaactggggataaacaa**ATG**

Translation start site
